# Evolutionary dynamics in the genome of ocular Chlamydia trachomatis strains from Northern Tanzania following mass drug administration

**DOI:** 10.1099/mgen.0.001431

**Published:** 2025-07-02

**Authors:** Ehsan Ghasemian, Athumani Ramadhani, Anna Harte, Elias Mafuru, Tamsyn Derrick, Tara Mtuy, Patrick Massae, Aiweda Malissa, Judith Breuer, Harry Pickering, Robin L. Bailey, David Mabey, Matthew J. Burton, Martin J. Holland

**Affiliations:** 1 Department of Clinical Research, London School of Hygiene & Tropical Medicine, London, UK; 2 Institute of Microbiology, Lausanne University Hospital, Lausanne, Switzerland; 3 Department of Ophthalmology, Kilimanjaro Christian Medical Centre, Moshi, Tanzania; 4 Division of Infection and Immunity, University College London, London, UK

**Keywords:** *Chlamydia trachomatis*, evolution, mass drug administration (MDA), Tanzania, trachoma, whole-genome sequencing (WGS)

## Abstract

Trachoma, caused by *Chlamydia trachomatis* (Ct), remains a leading cause of preventable infection-induced blindness worldwide. We conducted a 4-year longitudinal study in three trachoma-endemic villages in Northern Tanzania, tracking infection dynamics and factors influencing trachomatous scarring progression and persistence pre- and post-mass drug administration (MDA) interventions. We analysed 118 whole genomes of Ct originating from ocular swabs of children. Sample collection was conducted at 3-month intervals over 4 years, encompassing 15 timepoints. We studied Ct phylogeny and patterns of SNP accumulation within sequences in the Ct genotype A (CtA) and Ct genotype B (CtB) phylogenetic clades, with the association of clinical signs of trachoma and scarring progression. Of the samples analysed, 71 (60.2%) were identified as CtA and 47 (39.8%) as CtB. We observed a significant shift in genotype prevalence: CtB predominated in pre-MDA samples (36 out of 40, 90%), whilst CtA became the dominant genotype after the first MDA round (67 out of 78, 85.9%) (*P*<0.0001). Phylogenetic analysis revealed two distinct CtA clades: clade 1 (29 sequences) was primarily found pre-MDA and shared a common ancestor with Tanzanian CtA reference genomes, whilst clade 2 (42 sequences) emerged post-MDA and exhibited a characteristic ~6 kbp reduction in the plasticity zone (PZ). Similarly, CtB sequences formed two distinct clades, both sharing ancestry with a Tanzanian CtB reference genome. Notably, we identified variable genome reduction in the PZ (~4 and ~10 kbp) amongst 13 CtB sequences distributed across both clades. We documented a significant shift in Ct genotype distribution following the first round of MDA, characterized by the emergence of CtA strains with distinct genetic profiles compared to pre-MDA strains. The observed reductions in Ct genome size suggest ongoing evolutionary processes shaping these bacterial populations. Additional research is needed to understand the dynamic changes in Ct lineage composition before and after antibiotic interventions and to determine how variations in genome size influence Ct biology and its susceptibility to azithromycin treatment.

**Impact Statement**

Trachoma is a global health challenge caused by repeated infections of the eyes with *Chlamydia trachomatis* (Ct), leading to blindness if untreated. Whilst the World Health Organization introduced the SAFE (Surgery, Antibiotics, Facial cleanliness and Environmental improvement) strategy in 1998 to eliminate trachoma by 2020, this target has been extended to 2030. Despite great achievements that have been made through the implementation of the SAFE strategy, some countries, particularly those with marginalized communities, have not yet reached the disease elimination threshold, even after multiple rounds of mass drug administration (MDA) with azithromycin. In Northern Tanzania, where trachoma prevalence remains high in some regions, particularly amongst Maasai populations, a 4-year longitudinal study was initiated in 2012 to investigate factors influencing the persistence and progression of trachoma. The research utilized genomics to analyse the characteristics of Ct strains in these populations, aiming to understand why trachoma persists despite multiple MDA interventions and to explore the implications for future trachoma elimination efforts. By tracking Ct infection over 4 years, we discovered a remarkable shift in the prevalence of Ct genotypes following MDA interventions, revealing the emergence of the strains with distinct genetic characteristics. These findings represent the interplay between MDA programmes and pathogen evolution, highlighting the need for continuous genomic surveillance to inform effective trachoma control strategies.

## Data Summary

Tanzanian *Chlamydia trachomatis* sequencing data in the form of fastq.gz files used in this study can be accessed from the European Nucleotide Archive project accession PRJEB46956 (accessions ERR6491749–ERR6491834 and ERR9439579–ERR9439623).

## Introduction

Trachoma is a blinding disease caused by repeated infection of the conjunctiva with *Chlamydia trachomatis* (Ct) genotypes A, B, Ba and C [1,3], from which CtA and CtB are reported as the most prevalent [4,7]. Ct is the leading cause of bacterial sexually transmitted infections globally [8,9]. In 1998, the World Health Organization (WHO) introduced the SAFE (Surgery, Antibiotics, promotion of Facial cleanliness and Environmental improvement) strategy to eliminate trachoma as a public health problem by 2020 [10,12].Although great achievements have been made through the implementation of the SAFE strategy, the target date for the global elimination of blinding trachoma as a public health problem has been pushed back from 2020 to 2030 [13].

The A component of SAFE involves mass drug administration (MDA) utilizing azithromycin, a strategy successfully implemented in several endemic countries based on the presence of the clinical signs of inflammation [14,17]. By 2024, 18 endemic or hypoendemic countries had been validated for trachoma elimination [12,18]. However, more than 38 countries have not yet reached the disease elimination threshold, although they have received several rounds of MDA [12,18, 19]. Prior studies suggested that success in MDA in trachoma communities could be in part due to MDA coverage, interaction with untreated neighbouring communities, diversity of circulating strains pre- and post-MDA and Ct infection load as an indicator of the probability of re-emergence of infection, post-treatment [20,22]. Tanzania has successfully implemented different aspects of the SAFE strategy, but in some marginalized communities, mostly consisting of the Maasai populations in the northern regions, the prevalence of active trachoma has been found to be >30% in children aged 1–9 years, which is far greater than the WHO elimination threshold of <5% [23,24].

**Fig. 1.**
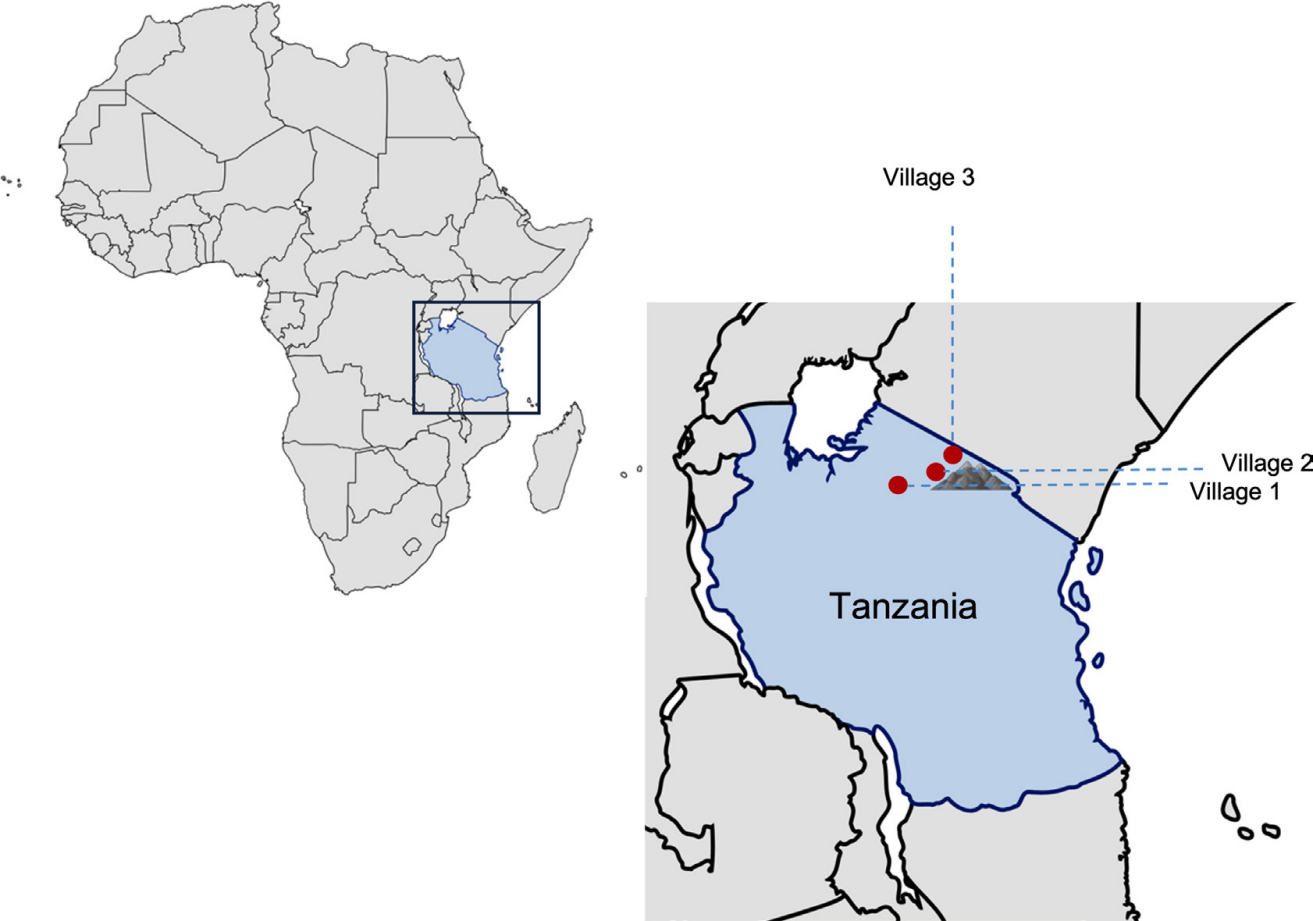
Geographical distribution of sampling sites in Tanzania. The study included three villages: village 1, located in the Longido district (Arusha region), and villages 2 and 3, situated in the Siha district (Kilimanjaro region). Figure created using BioRender.com.

**Fig. 2.**
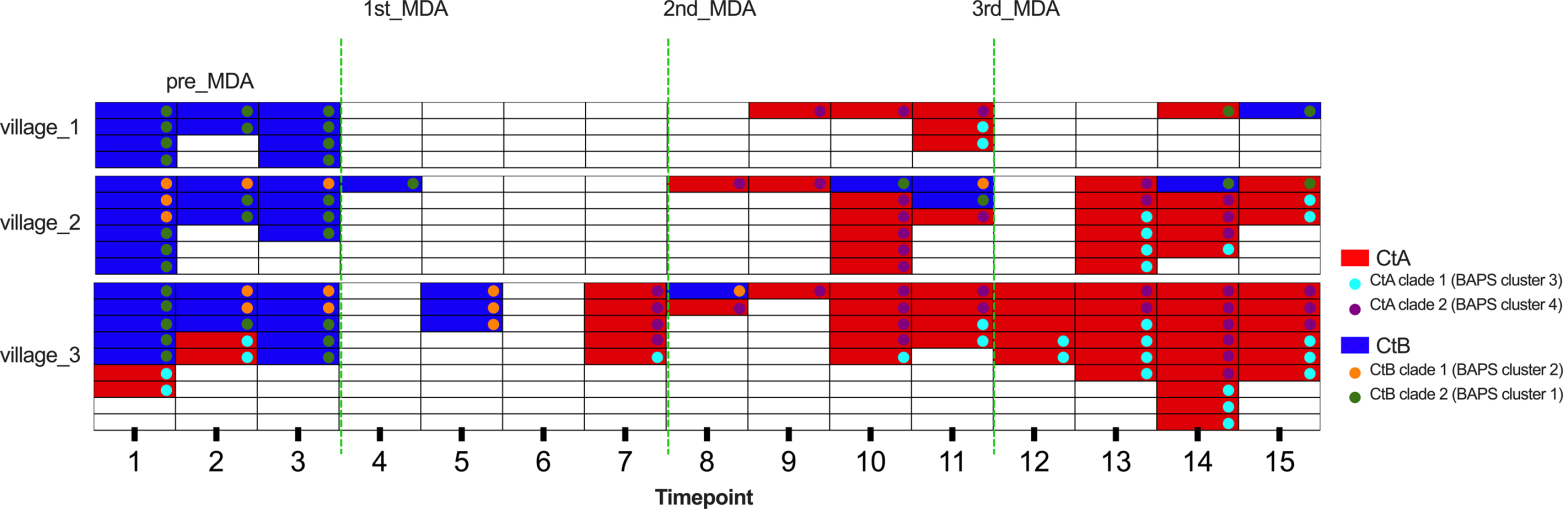
Temporal distribution of Ct genotypes and phylogenetic clades across Tanzanian villages throughout the study period. The timeline spans 15 sampling timepoints, with timepoints 1–3 representing pre-MDA samples and timepoints 4–15 representing post-MDA samples. Green dashed lines indicate the initiation of new MDA rounds. Squares indicate Ct genotype (A or B), whilst circles represent the specific phylogenetic clades and BAPS (Bayesian Analysis of Population Structure) clusters associated with CtA and CtB genomes.

In February 2012, we started a 4-year longitudinal study in three adjacent trachoma-endemic villages in northern regions of Tanzania with the aim of investigating the risk factors associated with scarring incidence and progression in Tanzanian children (Fig. 1) [21]. The cohort participants were followed up every 3 months, totalling 15 timepoints. Azithromycin MDA was administered by the study team according to WHO guidelines in August 2012, August 2013 and August 2014, following the third, seventh and eleventh timepoints, respectively, which provided us with pre-MDA (timepoints 1–3) and post-MDA (timepoints 4–15) samples (Fig. 2) [21,25]. Out of 118 Ct genomes in this study, 40 (33.9%) originated from pre-MDA, and 78 originated from one or more post-MDA treatment timepoints (Fig. 2). Here, we used genomics to explore genome characteristics of Ct strains circulating in the trachoma populations that, despite several rounds of MDA, did not reach the WHO elimination threshold.

## Methods

### Study design and sample collection

The study design and sample collection are described in detail by Ramadhani *et al*. [21]. In summary, we enrolled 666 children aged 6–10 years residing in three rural villages in Northern Tanzania (Fig. 1). Three communities are predominantly comprised of Maasai people [25]. Conducted from 2012 to 2016, the study involved sample collection at 3-month intervals, totalling 17 timepoints, with samples from timepoints 1–15 being included in our analysis (Fig. 2). The villages underwent three rounds of MDA with azithromycin following the third, seventh and eleventh timepoints. As part of the National Control of Neglected Tropical Diseases Programme, MDA with azithromycin, donated by Pfizer through the International Trachoma Initiative, was provided to all community members aged 6 months and older. A single-dose regimen of azithromycin (1 g for adults and 20 mg kg^−1^ for children) was administered annually over a 3-year period. For infants under 6 months of age, tetracycline eye ointment was supplied to their primary caregiver, with instructions for application to both eyes twice daily for 6 weeks.

At each sampling timepoint, trachoma clinical signs, namely, follicles, papillae and subtarsal conjunctival scarring, were evaluated and recorded on a scale of 0–3 [26]. This grading system can be translated to the WHO simplified trachoma grading system: where F2/F3 equates to trachomatous inflammation-follicular (TF), and P3 to trachomatous inflammation-intense (TI). Clinically active trachoma was defined as the presence of TF and/or TI [26,27]. We also considered that both P2 and P3 represent clinically significant trachomatous papillary-inflammation (TP) [28]. Trachomatous scarring (TS) scores collected at both the baseline and the concluding timepoint were used to categorize individuals into either scarring progressors or non-progressors, with any score increase denoting progression.

### Sample preparation, detection and genotyping of Ct

Dry swabs from the upper tarsal conjunctiva were collected and preserved at −80 °C until processing. DNA extraction and diagnostic quantitative PCR (qPCR) using chlamydial plasmid ORF 2 and a human endogenous control gene (RPP30) were carried out to ascertain Ct positivity, following previously described protocols [25,29]. Subsequently, the DNA samples underwent retesting for the Ct chromosomal *omc*B gene using a previously described droplet digital PCR assay [29]. For *omp*A genotyping and the exclusion of samples with evidence of mixed Ct genotype infections, the extracted DNA underwent a nested PCR, following the procedure outlined in Andreasen *et al*. [5]. Samples were sent to Source BioScience (Cambridge, UK) for Sanger sequencing in both directions [30]. The resulting *omp*A sequences were examined in Geneious (v2023.2.1) for ambiguous chromatogram signals indicative of potential mixed infections.

### Whole-genome sequencing and preparation of the sequences

Whole-genome sequencing (WGS) data were obtained directly from the Tanzanian samples with an *omc*B load ≥10 genome equivalents as previously described [7,31, 32]. Briefly, DNA baits spanning the length of the Ct genome were compiled by SureDesign and synthesized by SureSelectXT (Agilent Technologies, UK). The total DNA extracted from clinical samples was quantified, and carrier human genomic DNA was introduced to achieve a total input of 3 µg for library preparation. DNA was fragmented using a Covaris E210 acoustic focusing unit. Subsequent steps, including end-repair, non-templated addition of 3′-A, adapter ligation, hybridization, enrichment PCR and all post-reaction clean-up processes, were conducted following the SureSelectXT Illumina Paired-End Sequencing Library protocol (v1.4.1 Sept 2012). DNA was sequenced at University College London/University College London Hospitals Biomedical Research Pathogen Genomics Unit using Illumina paired-end technology (Illumina GAII or HiSeq 2000).

We utilized BBDuk (v38.84) to remove adapters, sequences shorter than 20 bp and those with a Phred quality score below 15 in 131 Ct whole-genome sequences [33]. Sequences were error-corrected and normalized utilizing BBNorm (v38.84) [34]. Subsequently, trimmed sequences were assessed using FastQC (v0.12.1) and MultiQC (v1.11) for ‘per base sequence quality’, ‘per sequence quality scores’, ‘per sequence GC content’, ‘per base N content’, sequence length distribution and the presence of overrepresented sequences and adaptors (Table S1, available in the online Supplementary Material) [35,36].

### Sequence mapping and annotation

Trimmed short reads were aligned to Ct reference strains A/2497 (GenBank FM872306; Tanzania, 2000) and B/TZ1A828/OT (GenBank FM872307; Tanzania, 1998) using BBMap v38.84, with a minimum read identity of 90% and read depth of 10× [33]. To ensure data quality, we classified WGS data as ‘high quality’ only if ≥95% of the nucleotide positions in the reference genome had a mean read depth of ≥10×. For samples meeting this threshold, consensus sequences were generated in FASTA format by applying majority-rule base calling to aligned reads. Strain classification (CtA vs. CtB) was determined based on the reference genome with the highest coverage (%Ref-Seq). Chromosomal genes were predicted using the annotated genome from Ct strain A/HAR13 (GenBank NC_007429) in Geneious with a minimum of 80% identity.

Antimicrobial resistance screening was done using staramr (v0.10.0), which is available at https://github.com/phac-nml/staramr to identify antimicrobial resistance genes in Tanzanian Ct whole genomes [37].

### Phylogenetic analysis

For the phylogenetic analyses of the Ct chromosome and plasmid, consensus FASTA sequences from 118 Tanzanian strains and 60 trachoma reference genomes [38,43], along with a T2 outgroup (Ct strain D_SotonD6) [38] (Table S2), were aligned using progressiveMauve (v2.4.0) [44]. To detect and remove recombination from the alignments, we used Gubbins (Genealogies Unbiased By recomBinations In Nucleotide Sequences) (v3.3.1) [45]. Predicted recombinant regions by Gubbins were masked by replacing affected positions with gaps ('-'). Subsequently, a phylogenetic tree was constructed employing RAxML (v8.2.11) and the general time reversible (GTR) model of evolution with a γ correction for among-site rate variation, utilizing four rate categories and conducting 200 bootstraps [46]. The resulting unrooted phylogenies were subsequently imported into Geneious for visualization and graphical presentation, where they were rooted using Ct strain D_SotonD6.

We applied hierarchical Bayesian Analysis of Population Structure (hierBAPS) to infer population clusters within the Tanzanian Ct whole genomes and plasmids post-recombination removal with Gubbins. hierBAPS (v1.1.4) was implemented with a maximum hierarchical depth of 2 and an initial population count of 50 for Ct whole genomes and 20 for Ct plasmids [47]. For visualization, a neighbour-joining phylogenetic tree was constructed from a pairwise distance matrix computed in ape (v5.8–1). Additionally, a maximum likelihood (ML) tree was inferred using phangorn (v2.12.1) with the GTR model, incorporating invariant sites and gamma distribution parameters. Cluster assignments from hierBAPS were mapped onto the phylogenetic trees, and visualizations were generated using ggtree (v3.12.0), with tip points colour-coded by cluster.

Ct *omp*A genes were extracted from the 118 Tanzanian and 61 reference genomes, and alignment was generated using MAFFT (v7.490) with a 200 PAM/K=2 scoring matrix [48,49]. PhyML (v3.3.20180621) was then applied to estimate ML phylogenies of aligned sequences, utilizing a GTR model of evolution and performing 200 bootstraps [50].

### Calling SNPs

To analyse the accumulation of SNPs within sequences in the CtA and CtB phylogenetic clades, we reconstructed a common ancestral sequence for each clade using ML ancestral state reconstruction. First, each of the CtA and CtB sequence sets was separately aligned, and recombination regions were removed using Gubbins. The ancestral sequences were then inferred using IQ-TREE (v4.0) with the best-fit nucleotide substitution model selected by ModelFinder (MFP option), and branch support was assessed using 1,000 ultrafast bootstrap replicates [51]. After phylogenetic reconstruction, empirical Bayesian ancestral state reconstruction was performed using the -asr flag in IQ-TREE to estimate the most probable nucleotide at each position of the ancestral nodes. A custom Python script was developed to extract the root node sequence (Node1) from the resulting ancestral probability tables (.state files), selecting the nucleotide with the highest posterior probability at each position in case of uncertainty.

SNPs were identified using the Geneious Prime variant/SNP caller tool by mapping short reads from CtA and CtB samples to their respective reconstructed ancestral sequences. Parameters included the following: minimum coverage threshold: 10×; minimum variant frequency: 90%; maximum variant *P*-value: 10^−6^ (Fisher’s exact test); strand-bias *P*-value threshold: 1×10^−5^, applied only when strand bias exceeded 65%. Moreover, we computed the proportion of nonsynonymous variations in the Tanzanian Ct genes using the Geneious variation/SNP caller tool.

We estimated the type of evolutionary pressure on the Tanzanian CtA and CtB whole genomes and the *omp*A gene by calculating the dN/dS ratio. For the detection of selection pressures acting on CDSs, we performed codon-based Z-tests of selection using mega11 software [52]. The analysis was conducted on aligned sequences using the modified Nei–Gojobori method with the Jukes–Cantor correction [53,54]. The test statistic (dN-dS) was calculated for each sequence pair, where dN and dS represent the number of nonsynonymous and synonymous substitutions per site, respectively [55,56]. The variance of the difference was computed using the bootstrap method with 200 replicates. The null hypothesis of neutral evolution (H_0_: dN=dS) was tested against the alternative hypothesis of purifying selection (H_1_: dN<dS). Statistical significance was established at *P*<0.05.

### Statistics

Association analyses were conducted in R (v4.2.1) using two response variable groups. The first used the follicle, papillae and scarring score, assessed at the time of the survey (*n*=118). Scarring and papillae were converted into binomial factors, as there were too few values above 1 to justify keeping them as ordinal variables; for scarring, score 0=0, and scores 1, 2=1. For papillae, scores 0, 1=0, and scores 2, 3=1. Follicle score was kept as an ordinal variable (scores 0, 1, 2, 3).

The second variable was scarring progression (*n*=78), which was determined by comparing clinical scores for scarring at the beginning and end of the study. This was a binomial factor, with 1 indicating that an individual had more severe scarring. The age at the beginning of the study was included in the scarring progression model to account for possible differences in scarring between younger and older children.

Binomial response variables were analysed using generalized linear models (R package lme4), with village tested as a potential random effect [57]. The variance of the random effect was examined, and the random effect was removed if the variance was <0.01. Univariate models were run to determine whether individual variables were significant, followed by multivariate models including all variables to test for the presence of any interactive effects. Gender, age and genomic genotype/clade (CtB, CtA clade 1 or CtA clade 2) were included as fixed effects, with age as a continuous variable with one unit equal to 1 year. The ordinal response was analysed using ordinal regression (v2023.12–4.1) including the same random and fixed effects [58]. The reference level was female for gender, and CtA clade 1 for genotype/clade. *P*-values have not been adjusted for multiple comparisons.

The distribution of Ct infection loads (log10) across CtA clade 1, CtA clade 2 and CtB groups was first tested for normality using the Shapiro–Wilk test. Differences in infection loads between groups were assessed using one-way ANOVA. Tukey’s multiple comparisons test was performed to identify specific differences between individual groups. Similarly, the distribution of mutation counts across CtA clade 1, CtA clade 2 and CtB groups was first tested for normality using the Shapiro–Wilk test. The relationship between Ct groups and number of mutations was assessed using the Kruskal–Wallis test. Dunn’s multiple comparisons test was performed to identify specific differences between individual groups. Statistical analysis was conducted using GraphPad Prism software (v10.1.1). Statistical significance was set at *P*<0.05.

## Results

### Study population and Ct WGS quality data

Of the 666 children initially enrolled in the study, 218 were excluded due to incomplete follow-up or missing outcome data, as previously described by Ramadhani *et al*. [21]. Antibiotic coverage of the remaining 448 participants included in the scarring progression analysis was 335 (79.2%) in 2012, 374 (83.5%) in 2013 and 338 (75.4%) in 2014. These 448 children were assessed at a median of 15 timepoints, and 219 tested positive for Ct at one or more timepoints. From 593 samples tested positive for Ct, a subset of 131 samples was of sufficient *omc*B load to ensure a high-quality sequence by direct WGS. Following quality control and mapping, 118 sequences were qualified for further analysis (Table S1). Seven participants provided samples at two sampling timepoints, and 104 participants provided samples at one sampling timepoint. From 118 sequences, 40 (33.9%) originated from pre-MDA, 9 (7.6%) post-first MDA, 27 (22.9%) after the second MDA and 42 (35.6%) following the third MDA (Fig. 2). The mean age of the study participants was 7.1 years (sd: 2.1), comprising 50 (45%) males and 61 (55%) females (Table S3).

Antimicrobial resistance screening using staramr did not detect any known antimicrobial resistance genes in the Tanzanian Ct strains. However, looking at the genes that may play a role in developing resistance to macrolides in Ct, we identified a mutation at position 526 (ATG>GTG, Met>Val) of the *rpl*D gene in all CtA sequences. Additionally, a mutation was observed at position 913 (GAT>AAT, Asp>Asn) of the *rlm*D gene in CtA clade 1 and CtB sequences.

### *omp*A diversity

We classified Tanzanian Ct isolates as genotypes A or B based on *omp*A genotyping using (i) phylogenetic reconstruction of *omp*A gene sequences derived from the Tanzanian Ct genomes (Fig. S1) and (ii) blast-n alignment of *omp*A sequences obtained via Sanger sequencing. As an additional genome-wide approach to evaluate consistency with *omp*A-based classification, we aligned short reads to Ct reference genomes A/2497 and B/TZ1A828/OT. This analysis revealed that 71 sequences (60.2%) exhibited higher reference genome coverage (%Ref-Seq) with the A/2497 reference genome, whilst 47 sequences (39.8%) showed higher reference genome coverage with the B/TZ1A828/OT reference genome (Table S3). We found complete concordance between traditional *omp*A-based genotyping results and these genome-wide similarity patterns, with all *omp*A-classified genotype A isolates showing higher similarity to the A/2497 reference (designated ‘CtA’) and all *omp*A-classified genotype B isolates showing higher similarity to the B/TZ1A828/OT reference (designated ‘CtB’).

Within the 40 pre-MDA sequences, four (10%) displayed the highest identity to CtA, whilst the remaining 36 (90%) showed the highest identity to CtB (*P*<0.0001) (Fig. 2). Conversely, in the 78 sequences obtained from post-MDA, 67 (85.9%) demonstrated the highest identity to CtA, and 11 (14.1%) to CtB (*P*<0.0001) (Fig. 2). Prior to the first MDA, all four (100%) CtA sequences were exclusively from village 3, whilst the 36 CtB sequences were distributed across all three villages (Fig. 2).

The ML reconstruction of the Tanzanian *omp*A sequences unveiled two distinct clades (Fig. S1). CtA sequences formed a cohesive group with Tanzanian genotype A reference strains, and CtB sequences grouped with the Tanzanian reference strain B/TZ1A828/OT. Within the Tanzanian CtB group, five sequences constituted a subgroup, which shared a substitution at position 1000 (G>A=A>T). Another substitution at position 484 (A>G= N>D) of CtB *omp*A in sample 15–058 led to an individual branch. Codon-based Z-tests of selection performed on the *omp*A gene revealed a non-significant trend toward purifying selection in both CtA and CtB (*P*>0.05).

### BAPS clustering and phylogeny of Ct chromosomes and plasmids

**Fig. 3.**
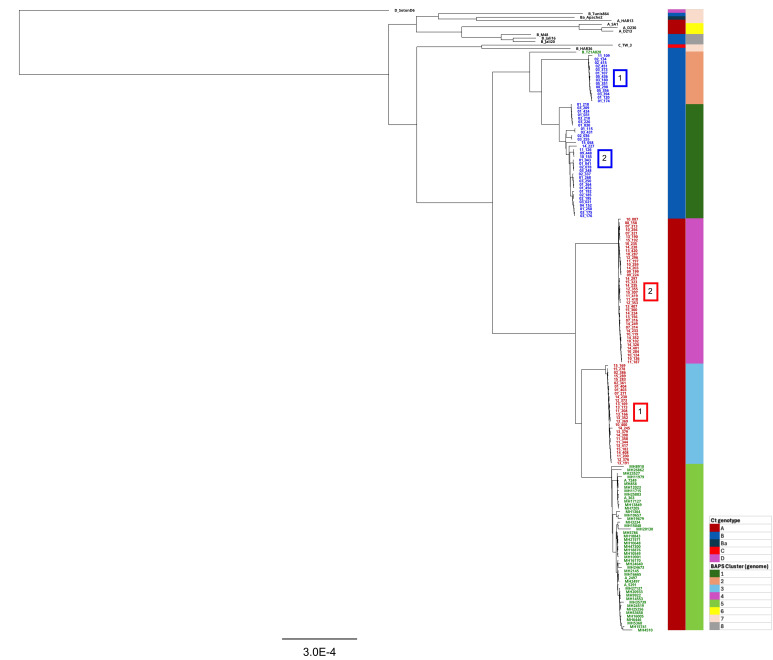
Ct chromosomal phylogeny. The analysis includes whole-genome sequences from 118 Tanzanian Ct strains, 60 trachoma reference genomes and Ct strain D_SotonD6 (T2) as an outgroup. Chromosomal sequences were aligned using progressiveMauve, and an ML phylogenetic tree was reconstructed with RAxML under the GTR model, incorporating a gamma (γ) correction for among-site rate variation with four rate categories and 200 bootstrap replicates. Whole-genome sequences of the Tanzanian samples positive for CtA are highlighted in red, whilst those positive for CtB are shown in blue. Tanzanian reference strains are marked in green, whereas other reference strains are in black. To improve the visualization of the phylogenetic relationships amongst Tanzanian samples from this study, the CtA and CtB clades that correspond to individual BAPS clusters are highlighted with numbered square boxes. The columns to the right of the tree indicate (**i**) the *omp*A genotype of each strain and (ii) BAPS clusters derived from whole-genome sequences. The scale bar represents the number of nucleotide substitutions per site.

BAPS clustering analysis of Ct whole genomes identified eight distinct population clusters across the dataset (Fig. S2). Tanzanian Ct sequences from this study were grouped into four BAPS clusters, with two corresponding to CtA (cluster 3: 29 sequences; cluster 4: 42 sequences) and two to CtB (cluster 1: 33 sequences; cluster 2: 14 sequences). Phylogenetic analysis of the Tanzanian Ct genomes from this study revealed two CtA clades corresponding to BAPS clusters 3 and 4, and two CtB clades corresponding to BAPS clusters 1 and 2, further supporting the population structure inferred through BAPS (Figs 3 and S2).

**Fig. 4.**
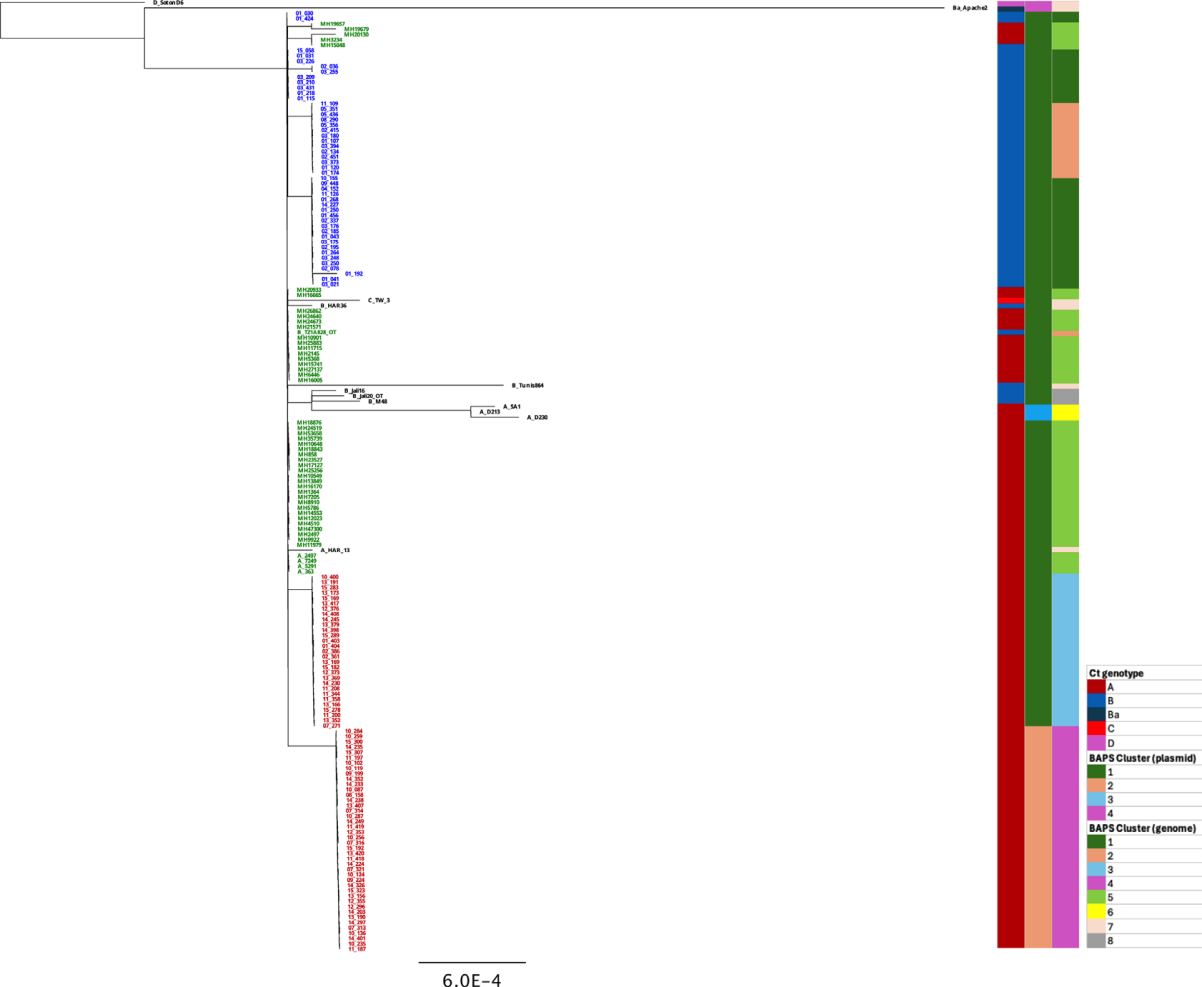
Ct plasmid phylogeny. The analysis includes plasmid sequences from 118 Tanzanian Ct strains, 60 trachoma reference genomes and Ct strain D_SotonD6 (T2) as an outgroup. Sequences alignment was done using progressiveMauve, and a phylogenetic tree was reconstructed with RAxML, incorporating the GTR model of evolution, incorporating a gamma (γ) correction for among-site rate variation with four rate categories and 200 bootstrap replicates. Tanzanian samples from this study testing positive for CtA are denoted in red, whilst those for CtB are indicated in blue. All reference strains originating from Tanzania are represented in green, and other reference strains are in black. The columns to the right of the tree indicate (i) the ompA genotype of each strain, (ii) BAPS clusters based on Ct plasmid sequences and (iii) BAPS clusters derived from whole-genome sequences. The scale bar represents the number of nucleotide substitutions per site.

Within the Tanzanian CtA clades, the first clade comprised 29 CtA sequences, sharing a common ancestor with 48 Tanzanian CtA reference genomes (Fig. 3). It is noteworthy that the only four sequences originating from pre-MDA fall within this lineage. The second clade consists of 42 CtA sequences, forming a distinct clade from the rest of the Tanzanian CtA sequences. Sequences from both Tanzanian CtB clades shared a common ancestor with the Tanzanian reference strain B/TZ1A828/OT (Fig. 4). The first clade consisted of 14 sequences, whilst the second, comprising 33 sequences, exhibited closer phylogenetic proximity to B/TZ1A828/OT and displayed longer branch lengths between some genomes, suggesting a greater within-clade genetic diversity.

BAPS clustering of Ct plasmids identified four distinct clusters (Fig. S3). Tanzanian plasmid sequences from this study were grouped into two of these clusters. A comparison with the phylogenetic distribution of Tanzanian Ct plasmid sequences showed that BAPS cluster 1 corresponds to the larger CtA plasmid clade, which includes 42 sequences, whilst BAPS cluster 2 encompasses all remaining CtA and CtB plasmid sequences (Figs 4 and S3) . Further comparison between Ct plasmid BAPS clusters and Ct genome BAPS clusters revealed that samples in plasmid BAPS cluster 1 correspond to those in genome BAPS cluster 3 (CtA genome clade 2) (Figs 3 and S3). In contrast, plasmid BAPS cluster 2 includes samples distributed across genome BAPS clusters 1, 2 and 4, which correspond to CtB genome clade 1, CtB genome clade 2 and CtA genome clade 1, respectively (Figs 3 and S3).

We compared the chromosome length of the Tanzanian CtA and CtB strains. The mean lengths of Tanzanian CtA and CtB chromosomes were 1,040,232 bp (sd: 3,626 bp) and 1,042,338 bp (sd: 3,936 bp), respectively. Comparing sequences from the two CtA clades revealed a reduction of ~6 kbp in sequences belonging to CtA clade 2 (length: 1,038,184 bp; sd: 529 bp) compared to the sequences from CtA clade 1 (length: 1,044,477 bp; sd: 287 bp) (Fig. 3). Furthermore, reductions of ~4 and ~10 kbp were observed in six and seven Tanzanian CtB chromosomes (length: 1,040,127 bp and sd: 667 bp and length: 1,033,695 bp and sd: 349 bp, respectively), compared to the rest of CtB sequences (length: 1,044,383 bp; sd: 697 bp). These reductions primarily stem from deletions in the plasticity zones (PZs). A detailed characterization of these deletions, including the specific genes affected, is provided in a later section: ‘comparative analysis of the plasticity zone’. We further investigated any evidence of genome reduction in 48 Tanzanian Ct reference strains (Table S2). Our investigation into these sequences revealed no disparities in genome size.

### Comparative analysis of the Tanzanian Ct strains

**Fig. 5.**
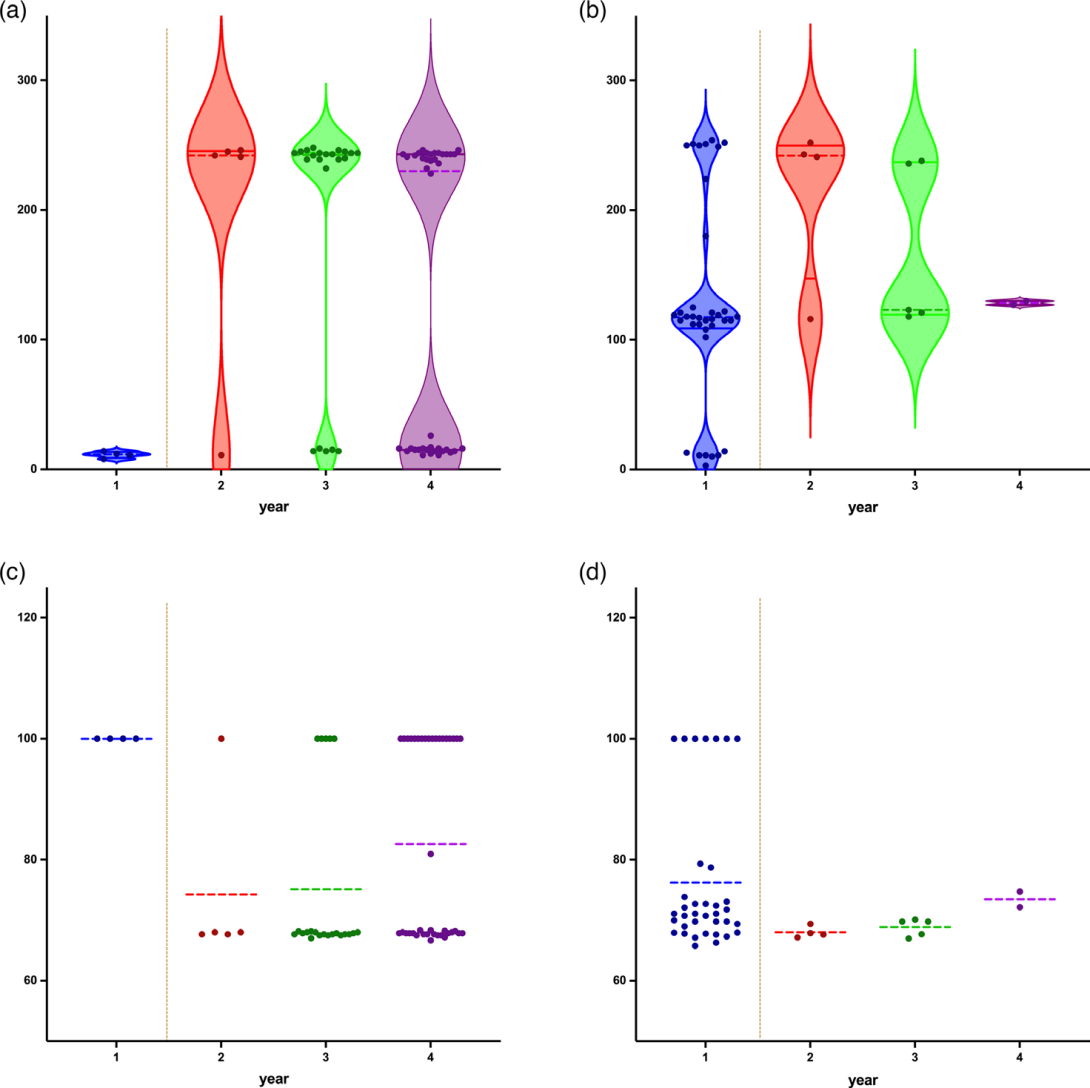
Distribution of SNPs and nonsynonymous mutation profiles in Tanzanian Ct genomes. (a, b) Analysis of SNP burden (total SNPs per genome) and (c, d) proportion of nonsynonymous SNPs in CtA and CtB genomes. Samples are grouped by collection year; the vertical yellow line delineates pre- and post-MDA timepoints. (a) Violin plot showing SNP burden distribution in CtA strains. (b) Violin plot showing SNP burden distribution in CtB strains. (c) Scatter plot displaying the proportion of nonsynonymous SNPs per genome in CtA strains. (d) Scatter plot displaying the proportion of nonsynonymous SNPs per genome in CtB strains. In panels (a) and (b), individual points represent SNP counts per whole-genome sequence. In panels (c) and (d), points indicate the percentage of nonsynonymous SNPs per genome.

Analysis of SNP events and the proportion of nonsynonymous mutations in CtA strains (mean SNPs=148.9, SD=112.7; mean nonsynonymous SNPs=80.7%, sd=15.8%) and CtB strains (mean SNPs=138.25, sd=77.8; mean nonsynonymous SNPs=74.64%, sd=11.1%) are presented in Fig. 5.

Further exploring CtA sequences demonstrated a division into two distinct groups based on SNP counts and proportion of nonsynonymous mutation: (i) a group of 29 sequences with a lower SNP burden but a higher proportion of nonsynonymous mutations (mean SNPs=14.3, sd=3; mean nonsynonymous SNPs=99.3%, sd=3.5%) and (ii) a group of 42 sequences with a higher number of SNPs but a lower proportion of nonsynonymous mutations (mean SNPs=241.85, sd=4.1; mean nonsynonymous SNPs=67.8%, sd=0.3%) (*P*<0.0001 for both comparisons) (Fig. 5). This is in line with the phylogenetic distribution of Tanzanian CtA genomes (Fig. 3), where CtA clade 1 comprises sequences from the first group with fewer SNPs, whilst CtA clade 2 consists of sequences from the second group with a greater SNP count.

CtB sequences could be divided into three groups based on the number of mutations. Fourteen CtB sequences with the highest number of SNP counts (mean SNPs=240.8, sd=19.4; mean nonsynonymous SNPs=67.4%, sd=0.65%) grouped together. These sequences correspond to the sequences forming CtB clade 1 genome phylogeny (Figs3  5). However, sequences comprising CtB clade 2 were divided into two distinct groups. The first group, consisting of seven sequences with a lower number of mutations (mean=10.4, sd=3.5; mean nonsynonymous SNPs=100%, sd=0%), corresponded to the smaller subclade within CtB clade 2. This subclade includes sequences 01_030, 01_031, 01_218, 01_424, 03_209, 03_210 and 03_226 (Figs3  5). The second group, comprising 26 sequences with a higher SNP count (SNP count, mean=117.5; mean nonsynonymous SNPs=71.7%, sd=2.6%), formed the larger subclade within CtB clade 2 (Figs3  5). There was a significant difference in the number of mutations and proportion of nonsynonymous mutations amongst CtB clade 1 and the two subclades within CtB clade 2 (*P*<0.0001 for both comparisons). Notably, seven sequences forming the smaller subclade within CtB clade 2 were only observed in pre-MDA timepoints.

CtA genomes showed evidence of purifying selection (*Z*=−2.02, *P*=0.045). When analysed by clade, CtA clade 1 exhibited strong purifying selection (*Z*=−3.86, *P*=0.00018), suggesting strict evolutionary conservation in this lineage. In contrast, CtA clade 2 showed a non-significant trend towards purifying selection (*Z*=−1.69, *P*=0.093), indicating potentially relaxed selective constraints. For CtB genomes, we observed significant purifying selection overall (*Z*=−4.42, *P*=0.00002); however, this pattern was inconsistent across clades. CtB clade 1 exhibited a non-significant trend towards purifying selection (*Z*=−1.33, *P*=0.19), whereas CtB clade 2 showed significant purifying selection (*Z*=−2.34, *P*=0.02).

### Comparative analysis of the PZ

**Fig. 6.**
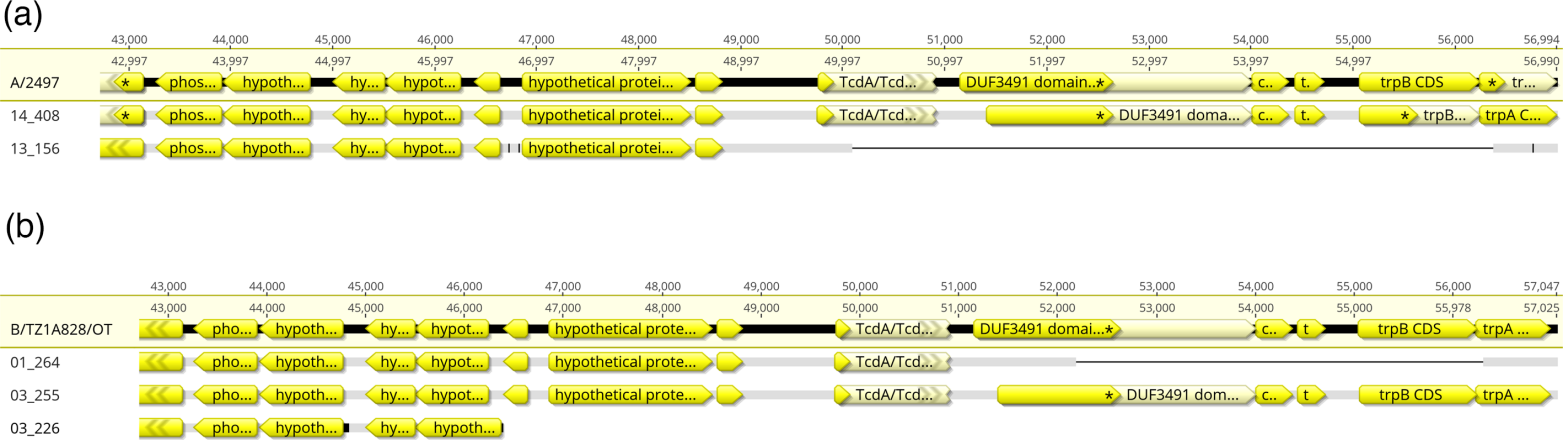
Comparative analysis of PZ reduction patterns in Tanzanian Ct strains. Alignments show CDS annotations across the terminal ~14 kbp region of the PZ for CtA (a) and CtB (b) strains. Each panel displays a reference strain (A/2497 for CtA; B/TZ1A828/OT for CtB) compared with representative Tanzanian strains exhibiting gene loss patterns. (a) CtA comparison showing sample 14_408 (clade 1) with intact PZ and sample 13_156 (clade 2) with evidence of gene loss (~6 kbp deletion). (b) CtB comparison showing sample 03_225 with intact PZ, and samples 01_264 and 03_226 with evidence of gene loss (~4 kbp and ~10 kbp deletion, respectively).

Comparative analysis of the Tanzanian Ct PZ by aligning 71 CtA sequences, including the reference strain A/2497, and 47 CtB sequences, including the reference strain B/TZ1A828/OT (Figs 6 and S4), revealed that two categories of the PZ for each Ct genotype consist of (i) those with an intact PZ (29 CtA clade 1 and 34 CtB) and (ii) those with evidence of genome reduction (42 CtA clade 2 and 13 CtB). In CtA clade 2, six genes were reduced encoding TcdA/TcdB catalytic glycosyltransferase (CT_166), DUF3491 domain-containing protein (CT_167), cytoadherence factor (CT_168), TrpR (CT_169), TrpB (CT_170) and TrpA (CT_171), resulting in a deletion of ~6 kbp (Fig. 6). Examining the PZ of CtA clade 1 sequences provided evidence indicating the presence of four truncated genes encoding TcdA/TcdB catalytic glycosyltransferase (CT_166), DUF3491 domain-containing protein (CT_167), TrpB (CT_170) and TrpA (CT_171) (Fig. 8). In 13 CtB sequences, the reduced region exhibited diversity in terms of size (~4 and 10 kbp) and deleted genes (Figs 6 and S4).

### Infection loads and clinical scores linked to Ct variants

In our analysis, CtA genome sequences formed two distinct phylogenetic clades, which were further supported by BAPS clustering, as well as phylogenetic and BAPS clustering analyses of CtA plasmid sequences (Figs3^ 4^, S2 and S3). These clades exhibited notable genomic differences, including variation in the number of accumulated mutations, percentage of nonsynonymous variations and genome size. Given these genetic and structural divergences, we subdivided CtA sequences based on their phylogenetic clades (CtA clade 1 and CtA clade 2) for further analysis of infection load and clinical scores.

**Fig. 7.**
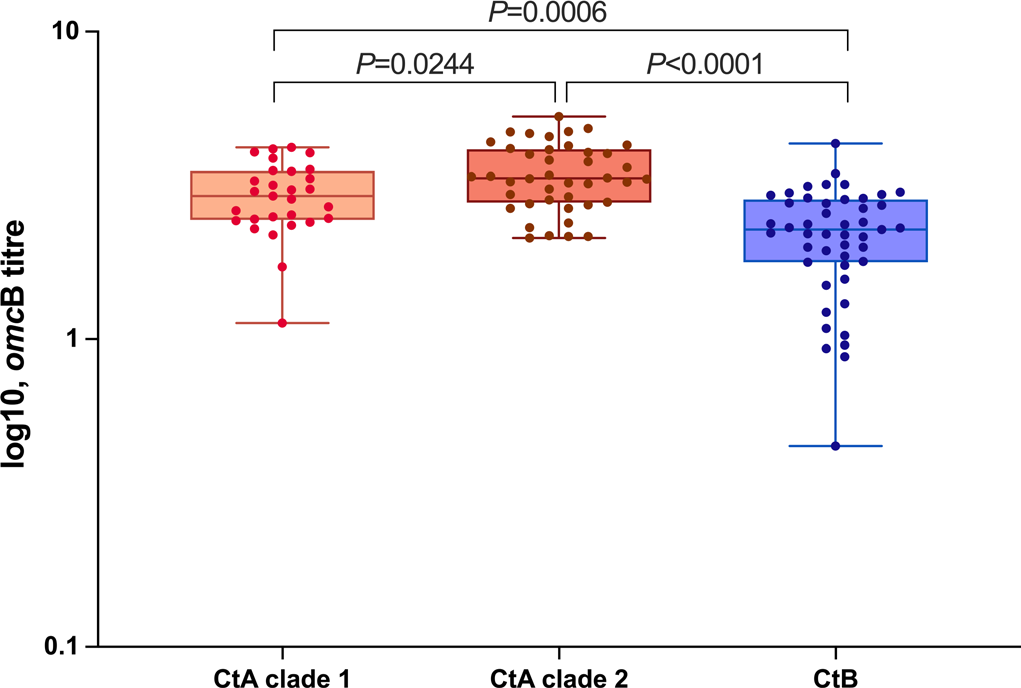
Ct *omc*B gene loads in Tanzanian samples. The dot plot shows the log10 Ct *omc*B titre in Tanzanian samples. The vertical lines indicate the error bars that express the sd, and the horizontal lines indicate the means.

Comparing the *omc*B copy numbers amongst the Tanzanian samples, we observed a significantly higher load of infection in CtA clade 2 compared to those from CtA clade 1 and CtB (*P*=0.0222 and *P*<0.0001, respectively). Moreover, we detected a significantly higher load of infection in CtA clade 1 samples compared with CtB samples (*P*<0.0001) (Fig. 7).

CtB was significantly positively associated with the presence of papillae in both univariate and multivariate models (*P*<0.05), and in the univariate model for scarring; the odds ratio indicated that with increasing age, there was a lower chance of having papillae or scarring (Table 1, Fig. S5). Age was significant in the univariate models for scarring and follicles (*P*<0.05). There were no significant variables for scarring progression in either the univariate or multivariate models (Table 1).

**Table 1.** Association analysis of age, gender and clade variables with three clinical score timepoint response variables and scarring progression in children in Tanzania from 2012 to 2016. Papillae, scarring at timepoint and scarring progression were treated as binomial values and analysed using generalized linear models, whilst follicle data were treated as an ordinal variable and analysed using ordinal regression

Response variable	Dependent variable	Level	Univariate odds ratio and 95% CI	Univariate *P*-value	Multivariate odds ratio and 95% CI	Multivariate *P*-value
Papillae	Age	Continuous	0.93 (0.42–1.07)	0.31	1.09 (0.92–1.31)	0.30
	Gender	Female	Reference	Reference	Reference	Reference
		Male	0.95 (0.46–1.98)	0.89	1.05 (0.48–2.28)	0.89
	Ct clade	CtA clade 1	Reference	Reference	Reference	Reference
		CtA clade 2	0.72 (0.27–2.00)	0.54	0.71 (0.25–1.95)	0.50
		CtB	2.64 (1.03–7.01)	0.04	3.57 (1.18–11.66)	0.02
Scarring	Age	Continuous	0.74 (0.58–0.92)	0.01	0.91 (0.768–1.19)	0.44
	Gender	Female	Reference	Reference	Reference	Reference
		Male	0.38 (0.10–1.16)	0.11	0.37 (0.09–1.21)	0.12
	Ct clade	CtA clade 1	Reference	Reference	Reference	Reference
		CtA clade 2	1.40 (0.13–30.99)	0.79	1.46 (0.13–32.41)	0.71
		CtB	10.71 (1.95–200.44)	0.03	8.08 (1.23–163.38)	0.06
Follicles	Age	Continuous	0.86 (0.76–0.97)	0.01	0.91 (0.78–1.06)	0.22
	Gender	Female	Reference	Reference	Reference	Reference
		Male	0.81 (0.42–1.58)	0.54	0.84 (0.43–1.66)	0.62
	Ct clade	CtA clade 1	Reference	Reference	Reference	Reference
		CtA clade 2	0.86 (0.36–2.03)	0.73	0.89 (0.37–2.12)	0.79
		CtB	2.25 (0.96–5.35)	0.06	1.67 (0.62–4.52)	0.31
Scarring progression	Age	Continuous	1.06 (0.85–1.33)	0.59	1.35 (1.00–1.86)	0.05
	Gender	Female	Reference	Reference	Reference	Reference
		Male	0.45 (0.12–1.50)	0.20	0.50 (0.12–1.77)	0.30
	Ct clade	CtA clade 1	Reference	Reference	Reference	Reference
		CtA clade 2	1.11 (0.18–8.93)	0.91	0.85 (0.12–7.31)	0.87
		CtB	3.18 (0.57–24.98)	0.21	7.87 (1.00–91.18)	0.06

## Discussion

We performed a comparative genomic analysis of ocular Ct strains circulating in three villages in Northern Tanzania, collected at 15 timepoints across 4 years, from which 3 were pre-MDA and 12 were post-MDA. From 118 Ct genomes collected during this longitudinal study, 71 were identified as genotype A and 47 as genotype B. Before MDA, most samples were classified as CtB (36 out of 40, 90%). However, CtA (67 out of 78, 85.9%) became the dominant strain in the population over time after the first round of MDA. After the first MDA, for up to three timepoints, no CtA genome was found in the population. However, at timepoint seven, we observed an influx of CtA strains with five positive samples in village 3, from which four were classified as a lineage of CtA that was not seen in our cohort prior to this timepoint. Comparative genomics analysis revealed distinct genomic features in this lineage in terms of genome size, and type and number of SNPs, distinguishing these strains from other Tanzanian CtA strains and previously published strains from Tanzania [40]. Notably, despite undergoing three rounds of MDA, unlike the other villages, village 3 Ct infection prevalence remained stubbornly high. This village is located within a district with historically higher levels of endemic trachoma [21]. Whilst multiple factors likely contribute to this persistence, including incomplete MDA coverage and potential reintroductions from neighbouring areas, Ct genetic dynamics may also play a role. Abdelsamed *et al*. suggested that genetic variation is a method by which Ct promotes chlamydial intracellular survival and evolves new phenotypes to evade host responses in a dynamic and niche-specific manner [59,60]. Finally, this study provides genetic evidence suggesting that clinical ocular Ct strains from Tanzania may have undergone gene reduction within the PZ region. This reduction appears to include genes encoding toxins and the TrpABR operon. This is important as gene families including cytotoxin and *trp* are attributed to explain most observed differences in chlamydial host specificity and tissue tropism [61,66].

The ML reconstruction of the Tanzanian CtA and CtB *omp*A sequences demonstrated two distinct clades. Within each clade, Ct *omp*A sequences from this study closely grouped with historical Tanzanian reference strains collected over a decade prior, showing minimal diversification over time. This limited *omp*A sequence variation over time was also noted in our previous work in The Gambia [31]. These observations contrast with prior reports suggesting that *omp*A often exhibits higher variability relative to other regions of the Ct genome [67,69]. The relative conservation of *omp*A sequences over time may reflect multiple factors, including the low overall substitution rate of the Ct genome reported previously by Hadfield *et al*. [70] for the L2b strain, possible functional constraints on major surface proteins in these endemic settings or regional differences in selective pressures [71,72]. The neutrality test results, indicating that Tanzanian *omp*A sequences did not significantly deviate from neutrality, further support the absence of strong diversifying selection on this gene in our study population.

The global phylogeny of Ct chromosomes revealed a distinctive distribution pattern of the Tanzanian CtA and CtB strains. The phylogenetic arrangement of CtA genomes presented two distinct clades: clade 1 comprising 29 sequences (40.8%), sharing a common ancestor with the Tanzanian CtA reference strain collected in 2000 and 2005, whilst clade 2 included 42 sequences (59.2%), suggesting the presence of a locally endemic clone. This phylogenetic divergence was accompanied by distinct evolutionary patterns. CtA clade 1 exhibited strong purifying selection, suggesting strict evolutionary conservation in this lineage. In contrast, CtA clade 2 displayed a non-significant trend towards purifying selection, suggesting potentially relaxed selective constraints. CtA clade 2 also exhibited a higher number of accumulated SNPs, a lower proportion of nonsynonymous mutations, evidence of genome reduction and a distinct plasmid phylogeny clade from the rest of Tanzanian plasmid sequences. The CtB genomes formed two clades, both sharing a common ancestor with the Tanzanian reference strain B/TZ1A828/OT, isolated in 1988. Within CtB clade 2, we identified a small subclade found exclusively in pre-MDA timepoints. This subclade exhibited fewer SNPs and a higher proportion of nonsynonymous mutations compared to the rest of CtB sequences. As with the CtA, we observed diverse evidence of purifying selection pressure across CtB clade 1 and CtB clade 2. In line with prior data, our findings may suggest the heterogeneous evolutionary pressures acting on different Ct genotypes [69,73]. Seth-Smith *et al*. [42] previously suggested that the inheritance of the Ct plasmid is closely linked to its corresponding chromosome. In our analysis, we identified a distinct phylogenetic clade for plasmids associated with genomes grouped within the CtA clade 2 lineage. In contrast, plasmids corresponding to CtA clade 1, CtB clade 1 and CtB clade 2 genomes grouped together into a single phylogenetic group. Further studies are needed to elucidate the coevolutionary dynamics between the Ct plasmid and chromosome.

Our findings revealed a shift in Ct dominance from genotype B pre-MDA to genotype A post-MDA. There are several likely explanations for this transition. First, the differing evolutionary constraints between CtA and CtB may have played a role. CtB sequences showed stronger purifying selection (*Z*=−4.42, *P*=0.00002) than CtA sequences (*Z*=−2.02, *P*=0.045), suggesting higher CtB fitness pre-MDA conditions. In contrast, the weaker purifying selection in CtA may have preserved greater genetic diversity, giving CtA a selective advantage under antibiotic pressure. Second, incomplete antibiotic coverage could have reshaped the population structure by creating bottlenecks, reducing genetic diversity through genetic drift and facilitating the rapid expansion of surviving strains [74,76]. This may explain the observed shift in the population structure, where CtA clade 2 strains, which were either absent or present at levels undetectable given our pre-MDA sample size, became dominant over CtA clade 1 strains in the post-MDA population. Similarly, this process may have contributed to the post-MDA disappearance of the smaller CtB clade 2 subclade, which comprised seven sequences. Third, the post-MDA lineage dynamics observed here might be due to the introduction of novel Ct strains into the sampled local population post-MDA.

Here, we provide evidence of genome reduction concentrated at the end of the PZ, removing six genes that are in common amongst CtA and CtB strains: CT_166–CT_171. Furthermore, in seven CtB strains, this reduction was expanded to four additional genes: CT_162–CT_165. In 2004, a study by Carlson *et al*. [77] indicated a genome reduction in the PZ (CT_162–CT_171) of a laboratory CtB strain: B/TW-5/OT, matching our observations in these seven CtB sequences. It is noteworthy to consider that in the majority of CtA clade 1 sequences with an intact PZ, CT_166, CT_167, CT_170/*trp*B and CT_171/*trp*A genes were truncated. Our observations may support the model of genome streamlining where non-functional genes are expected to be removed from the genome under a strong purifying selection effect [61,62, 78,80]. Previously, several studies demonstrated that obligate association with host tissues promotes genome reduction in bacteria [81,85]. Studies on Ct suggested that genome streamlining results from its co-evolution within the eukaryotic host [62,67, 86,88] and is facilitated by its ability to acquire essential metabolites from its host [89,92]. Our observations contradict prior speculations by Bohlin [93] and Andersson and Andersson [94] suggesting that the split into urogenital and ocular pathogroups is so recent that the consequences of relaxed selective pressure have not started to result in reduced genome sizes, and Ct likely reached the final stages of its reductive evolution.

We reviewed prior studies to understand how genome reduction could have potentially affected Ct characteristics. Moran [89] explained that in intracellular bacteria, selection has a favoured small genome size for the sake of growth efficiency or competitiveness within the host. Murray *et al*. [85] demonstrated a strong and negative association between bacterial genome size and pathogenicity, indicating that pathogenic species have smaller genomes than their non-pathogenic relatives [85]. Consequently, genome reduction could prove a useful marker of emerging and increasing pathogenicity in bacteria [85]. Prior studies illustrated that antibiotics exert effects beyond selecting for resistant variants [76,95], and sub- and non-lethal concentrations of antibiotics may enhance virulence and resistance evolution [76,95]. Here, we reported genome reduction in Tanzanian Ct strains, suggesting that the Ct genome is evolving. We recommend further investigations to understand (i) how this reduction in Ct genome has impacted its pathogenicity, infection biology and interaction with the host, and (ii) whether potential changes in Ct pathogenicity and infection biology because of genome reduction might have provided Ct with evolutionary advantages to overcome the MDA challenge.

There were no genes associated with antibiotic resistance in the Tanzanian Ct strains. However, we found non-synonymous mutations in the *rpl*D gene in CtA clade 1 and clade 2 and in the *rlm*D gene in CtA clade 1 and CtB sequences. Previously, it was suggested that Ct may develop resistance to macrolides via mutations in the 23S rRNA, *rpl*D and *rpl*V genes [96,99]. Additionally, the *rlm*D gene is responsible for methylating the 23S rRNA gene at position 1939 [100]. The main clinically relevant mechanism of resistance to macrolides has been reported as dimethylation of the 23S rRNA nucleotide A2058 located in the drug binding site [101,102]. Further studies are needed to confirm any link between these mutations and the development of a resistance phenotype towards azithromycin in Ct.

Our analysis revealed higher odds of all clinical signs and scarring progression related to CtB, with a significant association of TP. The odds ratio of TP increased when the multivariate analysis was run, suggesting that there may be an interaction between Ct genotype/clade and either age or gender. Similarly for scarring progression, the odds ratio almost doubled in the multivariate model compared to the univariate model. Previous studies have indicated a strong association between scarring progression and increased frequency of TP [25,28, 103, 104]. In 2019, we reported a gradual recovery in the prevalence of Ct infection in the Tanzanian cohort population post-MDA, whilst TP and TF prevalence remained low [21]. The dominance of CtB and CtA at pre- and post-MDA timepoints, respectively, as indicated by our current results, may explain these prior findings. Additionally, we found a significant correlation between CtB and TS in the univariate model, and close to significant in the multivariate model. Notably, individuals infected with CtA clade 2 exhibited a significantly higher infection load compared to those infected with CtA clade 1 and CtB. Previous studies have implicated more severe trachoma signs in individuals with higher infection loads [105,107]. Further studies on the induced immune responses by the Tanzanian CtA and CtB strains may explain why we are observing a lower likelihood of TP and scarring progression in CtA than in CtB despite higher loads of infection.

Our conclusions are constrained by the limited number of whole-genome sequences analysed. Deeper sampling across broader temporal and geographic scales could improve the power to differentiate between lineage introduction events and amplification of pre-existing diversity. The limited representation of CtA strains (4 out of 40, 10%) in pre-MDA timepoints, all originating from a single village, may have introduced a bias into the diversity of the sequences and pattern of SNP accumulation within the CtA sequences relative to CtB sequences. In analysing gene-level variation accumulation, our stringent variant-calling criteria minimize the risk of sequencing and mapping errors. However, we acknowledge that mis-mapping cannot be entirely excluded, particularly in repetitive or highly conserved genomic regions. A larger sample size may have provided greater insight into the overrepresentation of CtB strains at pre-MDA and CtA strains at post-MDA timepoints and of the potential link between the emergence of the CtA clade 2 and the initiation of MDA.

In summary, our findings demonstrated a notable shift in Ct genotype prevalence after the initial MDA round. The post-MDA lineage dynamics observed here might be due to (i) differential evolutionary constraints that conferred selective advantages to certain Ct strains under antibiotic pressure; (ii) incomplete antibiotic coverage, which may have reshaped the population structure by creating bottlenecks, reducing genetic diversity and enabling the rapid expansion of surviving strains; and (iii) the potential introduction of novel Ct strains into the Tanzanian population post-MDA. The observed evidence of ongoing genome reduction events in some of these strains may suggest that the intracellular adaptation of Ct is in progress. Here, we underscore the importance of studies to explore (i) the dynamic changes in Ct lineages before and after MDA interventions, (ii) the underlying factors driving genome reduction events, (iii) the implications of different genome reduction events on Ct biology and (iv) whether such events could impact Ct’s response to antibiotics.

## Supplementary material

10.1099/mgen.0.001431 Uncited Supplementary Material 1.

## Abbreviations

BAPS: Bayesian analysis of population structure
Ct: *Chlamydia trachomatis*
CtA: *Chlamydia trachomatis* genotype A
CtB: *Chlamydia trachomatis* genotype B
GTR: general time reversible
Gubbins: Genealogies Unbiased By recomBinations In Nucleotide Sequences
hierBAPS: hierarchical Bayesian Analysis of Population Structure
MDA: mass drug administration
ML: maximum likelihood
PZ: plasticity zone
qPCR: quantitative PCR
SAFE: Surgery, Antibiotics, Facial cleanliness and Environmental improvement
TF: trachomatous inflammation-follicular
TI: trachomatous inflammation-intense
TP: trachomatous papillary-inflammation
TS: trachomatous scarring
WGS: whole-genome sequencing
WHO: World Health Organization
