# Supplementary material for: Evolutionary dynamics in the genome of ocular Chlamydia trachomatis strains from Northern Tanzania following mass drug administration
Source: Microb Genom. 2025 Jul 2;11(7):001431. doi: 10.1099/mgen.0.001431 (PMC12222737; doi:10.1099/mgen.0.001431)
Supplement: Uncited Supplementary Material 1. [file mgen-11-01431-s001.pdf]

Supplementary data

Evolutionary dynamics in the genome of ocular *Chlamydia trachomatis* strains from Northern Tanzania following mass drug administration

Ehsan Ghasemian [1,2*], Athumani Ramadhani [3], Anna Harte [1], Elias Mafuru [3], Tamsyn Derrick [1], Tara Mtuy [3], Patrick Massae [3], Aiweda Malissa [3], Judith Breuer [4], Harry Pickering [1], Robin L. Bailey [1], David Mabey [1], Matthew J. Burton [1], Martin J. Holland [1]

[1] Department of Clinical Research, London School of Hygiene & Tropical Medicine, London, UK

[2] Institute of Microbiology, Lausanne University Hospital, Lausanne, Switzerland

[3] Department of Ophthalmology, Kilimanjaro Christian Medical Centre, Moshi, Tanzania

[4] Division of Infection and Immunity, University College London, London, UK

[*]Correspondence to: Ehsan.Ghasemian@lshtm.ac.uk

Supplementary Table 1. Whole genome sequencing statistics and data quality parameters for *Chlamydia trachomatis* strains.

Sample _id	ENA*-ERR accession	no._raw_reads	no._reads_post-trimming	per_base_sequence _quality	Ave._quality_per_read	%_gc	%_per_base_n_content	Mean_length (bp)	no._read_classified_as_Ct	Mean_read_depth	%_ref-seq	%_gc_after_mapping	Chromosome length (bp)
**01_030**	ERR9439579	2,642,066	675,726	>28	35	43.00%	0	150	549,341	77	98.60%	41.60%	1034269
**01_031**	ERR9439589	3,123,354	459,800	>28	40	41.80%	0	206	417,557	80	98.50%	41.40%	1033815
**01_041**	ERR9439580	2,468,342	766,008	>28	35	43.90%	0	149	568,067	79	99.50%	41.60%	1044547
**01_043**	ERR9439581	3,654,628	606,134	>28	35	42.10%	0	150	550,213	77	99.50%	41.50%	1044538
**01_107**	ERR9439582	2,596,858	770,574	>28	35	43.90%	0	149	566,506	79	99.60%	41.50%	1044681
**01_115**	ERR9439583	3,258,382	668,156	>28	35	43.00%	0	150	552,937	77	99.50%	41.50%	1044558
**01_120**	ERR9439584	2,252,596	537,288	>28	35	47.90%	0	156	184,993	26	96.40%	41.90%	1044533
**01_174**	ERR9439590	3,099,856	507,610	>28	35	46.60%	0	155	225,818	32	98.40%	41.70%	1044591
**01_192**	ERR9439585	3,008,548	623,838	>28	35	42.40%	0	150	563,339	79	99.50%	41.60%	1044577
**01_218**	ERR9439591	4,190,748	597,220	>28	35	45.00%	0	152	368,374	52	98.50%	41.80%	1033614
**01_250**	ERR9439592	3,761,122	504,516	>28	40	42.40%	0	205	425,796	82	99.50%	41.40%	1044591
**01_264**	ERR9439586	2,841,586	675,204	>28	36	42.70%	0	149	564,725	79	99.20%	41.50%	1040492
**01_268**	ERR9439594	2,738,360	492,506	>28	40	42.10%	0	203	431,015	82	99.20%	41.40%	1040772
**01_403**	ERR9439587	3,531,804	746,914	>28	35	43.80%	0	150	559,326	78	99.50%	41.60%	1044625
**01_404**	ERR9439588	3,661,116	779,126	>28	36	43.90%	0	149	571,310	79	99.50%	41.50%	1044579
**01_424**	ERR9439595	3,178,654	727,170	>28	36	43.40%	0	149	562,972	78	98.50%	41.50%	1033745
**01_456**	ERR9439596	3,058,964	616,404	>28	35	46.00%	0	153	308,310	43	99.00%	41.70%	1039337
**02_036**	ERR9439597	3,339,810	649,506	>28	36	46.40%	0	155	239,074	33	99.20%	41.60%	1041236
**02_078**	ERR9439598	3,107,868	782,788	>28	36	44.40%	0	151	483,439	68	99.50%	41.50%	1044546
**02_134**	ERR9439599	3,177,722	756,110	>28	36	44.70%	0	151	442,452	62	99.50%	41.50%	1044590
**02_185**	ERR9439601	3,466,488	672,854	>28	37	43.10%	0	151	511,179	72	99.50%	41.40%	1044420
**02_195**	ERR9439602	2,460,830	630,038	>28	36	44.00%	0	151	408,053	57	99.50%	41.40%	1044591
**02_337**	ERR9439604	3,342,474	732,506	>28	36	43.70%	0	151	507,576	71	99.20%	41.40%	1040565
**02_361**	ERR9439605	3,243,910	778,294	>28	36	44.40%	0	151	485,558	68	99.50%	41.50%	1044585
**02_386**	ERR9439606	4,610,112	1,055,292	>28	36	45.50%	0	151	486,211	68	99.50%	41.50%	1044571
**02_415**	ERR9439608	3,258,758	810,568	>28	36	44.70%	0	151	493,109	69	99.50%	41.50%	1044561
**02_451**	ERR9439609	3,209,890	783,732	>28	36	44.30%	0	151	507,843	71	99.50%	41.50%	1044542
**03_021**	ERR9439610	3,362,140	823,968	>28	36	44.60%	0	150	498,918	70	99.50%	41.50%	1044588
**03_175**	ERR9439611	3,074,158	761,694	>28	36	45.40%	0	152	392,000	55	99.50%	41.60%	1044533
**03_176**	ERR9439612	3,414,178	825,400	>28	36	44.50%	0	151	506,262	71	99.60%	41.50%	1044701
**03_180**	ERR9439613	3,494,540	822,694	>28	36	44.40%	0	151	503,395	71	99.60%	41.50%	1044637
**03_209**	ERR9439614	2,937,266	763,624	>28	36	44.60%	0	151	449,184	63	98.50%	41.50%	1033686
**03_210**	ERR9439615	3,164,034	804,946	>28	36	45.20%	0	151	413,446	58	98.50%	41.60%	1033654
**03_226**	ERR9439616	2,969,106	598,186	>28	36	45.70%	0	154	272,843	38	98.40%	41.60%	1033083
**03_248**	ERR9439617	3,151,454	771,574	>28	36	44.90%	0	151	444,692	62	99.50%	41.50%	1044582
**03_250**	ERR9439618	3,312,910	704,470	>28	36	46.20%	0	154	285,212	40	99.10%	41.60%	1039471
**03_255**	ERR9439619	3,271,076	763,576	>28	36	44.00%	0	151	513,624	72	99.50%	41.50%	1044522
**03_373**	ERR9439621	3,547,822	808,724	>28	37	44.20%	0	151	515,016	72	99.50%	41.40%	1044550
**03_394**	ERR9439622	3,172,094	769,304	>28	36	45.00%	0	152	430,629	61	99.50%	41.60%	1044523

**03_431**	ERR9439623	3,166,306	703,886	>28	36	45.40%	0	153	343,942	48	99.50%	41.50%	1044444
**04_152**	ERR6491821	3,357,490	862,090	>28	37	41.50%	0	151	445,374	62	99.50%	41.80%	1044443
**05_351**	ERR6491822	3,513,372	1,550,862	>28	38	40.90%	0	152	277,565	38	98.30%	42.30%	1044591
**05_356**	ERR6491823	3,769,340	1,580,430	>28	38	41.10%	0	149	469,876	65	99.50%	41.90%	1044371
**05_436**	ERR6491824	3,445,894	662,804	>28	37	42.10%	0	151	477,179	67	99.50%	41.70%	1044556
**07_271**	ERR6491825	3,451,398	1,158,560	>28	38	41.10%	0	148	514,237	71	99.50%	41.70%	1044544
**07_313**	ERR6491826	3,490,414	755,584	>28	38	42.30%	0	147	519,040	71	98.90%	41.70%	1038301
**07_314**	ERR6491827	3,424,286	813,184	>28	38	42.20%	0	148	512,894	71	98.90%	41.60%	1038311
**07_316**	ERR6491828	3,616,414	588,184	>28	37	41.80%	0	149	500,153	69	98.90%	41.60%	1038323
**07_321**	ERR6491829	3,443,714	761,108	>28	37	42.10%	0	149	507,493	70	98.90%	41.70%	1038328
**08_158**	ERR6491830	4,170,168	664,020	>28	38	41.70%	0	147	526,808	72	99.00%	41.70%	1038422
**08_290**	ERR6491831	3,592,028	430,852	>28	38	43.70%	0	150	258,615	35	99.30%	42.60%	1042026
**09_199**	ERR6491832	3,650,714	628,082	>28	37	42.20%	0	149	501,919	70	99.00%	41.60%	1038346
**09_224**	ERR6491833	3,573,760	602,874	>28	37	42.00%	0	149	504,549	70	98.90%	41.60%	1038347
**09_448**	ERR6491834	3,608,886	589,100	>28	37	41.80%	0	150	491,910	69	99.50%	41.60%	1044576
**10_087**	ERR6491749	2,895,784	1,658,530	>28	37	41.70%	0	151	409,272	57	98.70%	42.10%	1035893
**10_102**	ERR6491750	1,951,312	491,430	>28	37	41.50%	0	152	468,802	66	98.90%	41.50%	1038128
**10_119**	ERR6491752	3,892,978	1,491,282	>28	38	41.20%	0	148	489,150	67	98.90%	42.00%	1038048
**10_124**	ERR6491753	3,562,536	723,652	>28	37	42.80%	0	148	478,681	66	98.90%	42.00%	1038342
**10_136**	ERR6491754	4,004,308	545,876	>28	37	41.60%	0	148	496,644	69	98.90%	41.60%	1038329
**10_155**	ERR6491755	3,386,192	1,297,094	>28	38	43.10%	0	145	542,883	74	99.50%	41.70%	1044469
**10_235**	ERR6491757	3,901,566	631,094	>28	37	42.70%	0	149	446,483	62	98.90%	42.10%	1038230
**10_256**	ERR6491758	3,969,240	691,054	>28	39	42.50%	0	141	518,282	71	98.90%	41.80%	1038336
**10_259**	ERR6491759	4,796,040	574,822	>28	37	41.60%	0	148	507,443	70	98.90%	41.60%	1038316
**10_284**	ERR6491760	5,267,886	624,450	>28	38	41.40%	0	148	569,097	75	98.90%	41.40%	1038288
**10_287**	ERR6491761	3,159,182	573,912	>28	37	41.80%	0	148	517,227	71	98.90%	41.70%	1038325
**10_400**	ERR6491762	1,430,334	585,484	>28	36	41.90%	0	152	459,265	65	99.00%	41.60%	1038619
**11_109**	ERR6491763	2,716,518	2,254,660	>28	37	42.80%	0	151	394,670	55	96.80%	42.00%	1044533
**11_126**	ERR6491764	3,526,144	817,536	>28	37	42.10%	0	150	481,518	67	99.50%	41.80%	1044563
**11_187**	ERR6491765	3,731,216	626,768	>28	37	41.80%	0	149	502,399	70	98.90%	41.60%	1038338
**11_197**	ERR6491767	4,915,894	581,232	>28	37	41.60%	0	149	494,545	68	99.00%	41.50%	1038347
**11_200**	ERR6491768	3,612,254	2,058,824	>28	38	40.90%	0	149	465,644	65	99.50%	41.90%	1044069
**11_208**	ERR6491769	4,617,244	648,594	>28	38	41.90%	0	148	516,580	71	99.50%	41.60%	1044597
**11_344**	ERR6491770	3,575,218	541,930	>28	37	41.90%	0	150	491,101	68	99.50%	41.60%	1044554
**11_358**	ERR6491771	3,692,316	1,093,718	>28	38	41.70%	0	149	505,448	70	99.50%	41.60%	1044578
**11_418**	ERR6491773	5,134,072	573,638	>28	37	41.60%	0	149	492,054	68	98.90%	41.50%	1038320
**11_419**	ERR6491774	4,866,036	580,276	>28	37	41.70%	0	148	499,335	69	98.90%	41.50%	1038318
**12_296**	ERR6491775	4,238,662	590,588	>28	38	41.60%	0	148	514,866	71	98.90%	41.60%	1038340
**12_353**	ERR6491776	3,418,438	869,746	>28	38	41.80%	0	148	514,704	71	99.00%	41.80%	1038371
**12_355**	ERR6491777	3,718,010	1,002,168	>28	38	40.90%	0	148	516,033	71	98.90%	41.60%	1038279
**12_373**	ERR6491778	3,573,820	954,956	>28	38	41.80%	0	148	509,506	70	99.50%	42.10%	1044618
**12_376**	ERR6491779	3,677,412	636,640	>28	37	42.90%	0	150	344,813	47	99.50%	42.40%	1044257
**13_156**	ERR6491780	4,031,674	1,420,156	>28	38	41.30%	0	150	458,172	64	98.90%	41.90%	1038266

**13_166**	ERR6491781	3,712,964	690,910	>28	37	42.50%	0	149	496,817	69	99.50%	41.60%	1044470
**13_169**	ERR6491782	6,620,336	1,380,544	>28	38	41.40%	0	149	504,638	70	99.50%	41.60%	1044550
**13_173**	ERR6491783	3,011,084	772,106	>28	37	41.60%	0	150	495,724	69	99.50%	41.60%	1044572
**13_190**	ERR6491784	3,430,824	675,320	>28	37	42.40%	0	151	325,105	45	98.90%	42.30%	1037740
**13_191**	ERR6491785	3,870,076	1,696,694	>28	38	40.80%	0	152	265,503	37	98.30%	42.40%	1031221
**13_352**	ERR6491786	3,689,440	545,430	>28	38	41.80%	0	149	501,899	69	99.50%	41.50%	1044562
**13_369**	ERR6491787	3,201,530	754,766	>28	37	41.90%	0	149	500,507	70	99.50%	41.70%	1044534
**13_379**	ERR6491788	3,740,480	639,456	>28	37	42.10%	0	148	514,325	71	99.60%	41.60%	1044663
**13_407**	ERR6491789	3,452,582	678,530	>28	37	41.70%	0	149	499,866	69	98.90%	41.60%	1038319
**13_417**	ERR6491790	3,660,764	573,172	>28	37	42.00%	0	150	493,605	69	99.50%	41.60%	1044588
**13_420**	ERR6491791	3,525,790	660,090	>28	37	42.30%	0	150	489,552	68	98.90%	41.70%	1038308
**14_203**	ERR6491792	3,631,686	1,318,758	>28	38	43.20%	0	145	550,952	75	98.90%	41.80%	1038307
**14_224**	ERR6491793	3,704,394	2,413,696	>28	38	41.40%	0	149	477,171	66	98.90%	41.90%	1037546
**14_227**	ERR6491794	3,670,806	596,550	>28	37	42.90%	0	149	432,372	60	99.50%	42.10%	1044544
**14_230**	ERR6491795	4,061,516	563,302	>28	38	41.80%	0	148	507,944	70	99.50%	41.60%	1044560
**14_233**	ERR6491796	3,469,864	679,240	>28	37	42.50%	0	148	509,216	70	98.90%	41.90%	1038322
**14_235**	ERR6491797	3,216,636	669,074	>28	37	42.40%	0	148	514,588	71	98.90%	41.70%	1038341
**14_238**	ERR6491798	3,250,798	674,954	>28	38	41.90%	0	148	510,980	70	98.90%	41.60%	1038352
**14_245**	ERR6491799	3,666,412	2,593,064	>28	38	41.50%	0	149	347,479	48	97.90%	42.20%	1026947
**14_249**	ERR6491800	3,546,144	671,710	>28	38	41.70%	0	148	511,478	70	98.90%	41.60%	1038228
**14_297**	ERR6491802	4,061,676	1,083,002	>28	38	41.60%	0	148	359,312	49	98.80%	42.50%	1036714
**14_326**	ERR6491803	3,496,086	621,196	>28	37	42.10%	0	148	513,807	71	99.00%	41.70%	1038379
**14_352**	ERR6491806	3,960,548	711,926	>28	38	42.40%	0	146	523,909	71	99.00%	42.00%	1038350
**14_398**	ERR6491807	3,885,652	1,254,472	>28	38	42.10%	0	148	506,472	70	99.50%	41.80%	1044447
**14_401**	ERR6491808	3,325,146	789,710	>28	37	41.70%	0	150	498,778	69	98.90%	41.70%	1038296
**14_408**	ERR6491809	3,935,542	754,864	>28	38	42.00%	0	147	531,856	73	99.50%	41.80%	1044554
**15_058**	ERR6491810	3,211,600	803,786	>28	37	42.50%	0	149	506,163	70	99.50%	41.60%	1044559
**15_169**	ERR6491811	3,783,086	551,542	>28	38	41.80%	0	148	508,860	70	99.50%	41.60%	1044552
**15_182**	ERR6491812	3,770,456	565,646	>28	37	42.60%	0	153	248,386	34	99.40%	42.40%	1043197
**15_192**	ERR6491813	3,594,940	1,956,896	>28	38	40.30%	0	150	393,975	55	98.90%	42.00%	1037410
**15_278**	ERR6491815	2,984,712	1,296,150	>28	37	42.20%	0	149	499,349	69	99.50%	41.60%	1044510
**15_283**	ERR6491816	3,553,660	1,039,632	>28	37	41.90%	0	150	495,660	69	99.50%	41.60%	1044501
**15_289**	ERR6491817	4,087,624	589,920	>28	38	41.90%	0	148	511,277	71	99.50%	41.60%	1044573
**15_300**	ERR6491818	3,957,330	777,306	>28	37	42.70%	0	149	468,591	65	98.90%	42.00%	1038226
**15_307**	ERR6491819	3,529,082	713,180	>28	37	42.40%	0	150	496,551	69	99.10%	41.60%	1039599
**15_323**	ERR6491820	4,559,458	1,937,788	>28	38	41.30%	0	149	434,838	60	98.90%	42.00%	1037669

*ENA: European nucleotide archive

Supplementary Table 2. GenBank accession numbers and associated metadata for *Chlamydia trachomatis* reference genomes used in this study.

Isolate name	Lineage	Genotype	Country	Year	Source	ENA*-ERR accession	ENA*-ERS accession	Genome-accession	Reference
**A_2497**	ocular	A	Tanzania	2000	ocular	-	-	FM872306	Harris 2012
**A_363**	ocular	A	Tanzania	2000	ocular	ERR034213	ERS017900	-	Harris 2012
**A_5291**	ocular	A	Tanzania	2000	ocular	ERR034214	ERS017901	-	Harris 2012
**A_7249**	ocular	A	Tanzania	2000	ocular	ERR034215	ERS017902	-	Harris 2012
**A_D213**	ocular	A	Gambia	2001	ocular	ERR175652	ERS177838	-	Andersson 2016
**A_D230**	ocular	A	Gambia	2001	ocular	ERR111554	ERS075177	-	Hadfield 2017
**A_HAR13**	ocular	A	Egypt	1958	conjunctiva		-	CP000051	Carlson 2005
**A_MH10549**	ocular	A	Tanzania	2000	ocular	ERR175582	ERS177738	-	Harris 2012
**A_MH10648**	ocular	A	Tanzania	2000	ocular	ERR175583	ERS177739	-	Harris 2012
**A_MH10901**	ocular	A	Tanzania	2000	ocular	ERR175584	ERS177740	-	Harris 2012
**A_MH11715**	ocular	A	Tanzania	2000	ocular	ERR175585	ERS177741	-	Harris 2012
**A_MH11979**	ocular	A	Tanzania	2000	ocular	ERR175586	ERS177742	-	Harris 2012
**A_MH12023**	ocular	A	Tanzania	2000	ocular	ERR175565	ERS177721	-	Harris 2012
**A_MH1364**	ocular	A	Tanzania	2000	ocular	ERR175575	ERS177731	-	Harris 2012
**A_MH13849**	ocular	A	Tanzania	2000	ocular	ERR175566	ERS177722	-	Harris 2012
**A_MH14553**	ocular	A	Tanzania	2000	ocular	ERR175567	ERS177723	-	Harris 2012
**A_MH15048**	ocular	A	Tanzania	2000	ocular	ERR175587	ERS177743	-	Harris 2012
**A_MH15741**	ocular	A	Tanzania	2000	ocular	ERR175588	ERS177744	-	Harris 2012
**A_MH16005**	ocular	A	Tanzania	2000	ocular	ERR175568	ERS177724	-	Harris 2012
**A_MH16170**	ocular	A	Tanzania	2000	ocular	ERR175589	ERS177745	-	Harris 2012
**A_MH16665**	ocular	A	Tanzania	2000	ocular	ERR175590	ERS177746	-	Harris 2012
**A_MH17127**	ocular	A	Tanzania	2000	ocular	ERR175591	ERS177747	-	Harris 2012
**A_MH18843**	ocular	A	Tanzania	2000	ocular	ERR175569	ERS177725	-	Harris 2012
**A_MH18876**	ocular	A	Tanzania	2000	ocular	ERR175592	ERS177748	-	Harris 2012
**A_MH19657**	ocular	A	Tanzania	2000	ocular	ERR175570	ERS177726	-	Harris 2012
**A_MH19679**	ocular	A	Tanzania	2000	ocular	ERR175593	ERS177749	-	Harris 2012
**A_MH20130**	ocular	A	Tanzania	2000	ocular	ERR175594	ERS177750	-	Harris 2012
**A_MH20933**	ocular	A	Tanzania	2000	ocular	ERR175595	ERS177751	-	Harris 2012
**A_MH2145**	ocular	A	Tanzania	2000	ocular	ERR175561	ERS177717	-	Harris 2012
**A_MH21571**	ocular	A	Tanzania	2000	ocular	ERR175596	ERS177752	-	Harris 2012
**A_MH23527**	ocular	A	Tanzania	2000	ocular	ERR175571	ERS177727	-	Harris 2012
**A_MH24519**	ocular	A	Tanzania	2000	ocular	ERR175597	ERS177753	-	Harris 2012
**A_MH24640**	ocular	A	Tanzania	2000	ocular	ERR175598	ERS177754	-	Harris 2012
**A_MH24673**	ocular	A	Tanzania	2000	ocular	ERR175572	ERS177728	-	Harris 2012
**A_MH2497**	ocular	A	Tanzania	2000	ocular	ERR175562	ERS177718	-	Harris 2012
**A_MH25256**	ocular	A	Tanzania	2000	ocular	ERR175573	ERS177729	-	Harris 2012
**A_MH25883**	ocular	A	Tanzania	2000	ocular	ERR175599	ERS177755	-	Harris 2012
**A_MH26862**	ocular	A	Tanzania	2000	ocular	ERR175600	ERS177756	-	Harris 2012
**A_MH27137**	ocular	A	Tanzania	2000	ocular	ERR175601	ERS177757	-	Harris 2012
**A_MH3234**	ocular	A	Tanzania	2000	ocular	ERR175576	ERS177732	-	Harris 2012
**A_MH34496**	ocular	A	Tanzania	2000	ocular	ERR175602	ERS177758	-	Harris 2012
**A_MH35739**	ocular	A	Tanzania	2000	ocular	ERR175603	ERS177759	-	Harris 2012
**A_MH4510**	ocular	A	Tanzania	2000	ocular	ERR175577	ERS177733	-	Harris 2012
**A_MH47300**	ocular	A	Tanzania	2000	ocular	ERR175574	ERS177730	-	Harris 2012
**A_MH53658**	ocular	A	Tanzania	2000	ocular	ERR175604	ERS177760	-	Harris 2012
**A_MH5368**	ocular	A	Tanzania	2000	ocular	ERR175563	ERS177719	-	Harris 2012
**A_MH5786**	ocular	A	Tanzania	2000	ocular	ERR175564	ERS177720	-	Harris 2012
**A_MH6446**	ocular	A	Tanzania	2000	ocular	ERR175578	ERS177734	-	Harris 2012
**A_MH7205**	ocular	A	Tanzania	2000	ocular	ERR175579	ERS177735	-	Harris 2012
**A_MH858**	ocular	A	Tanzania	2000	ocular	ERR175560	ERS177716	-	Harris 2012
**A_MH8910**	ocular	A	Tanzania	2000	ocular	ERR175580	ERS177736	-	Harris 2012
**A_SA1**	ocular	A	Saudi Arabia	1957	ocular	ERR558498	ERS177777	-	Harris 2012
**B_HAR36**	ocular	B	Saudi Arabia	1969	ocular	ERR12253486	-		Ghasemian 2024
**B_Jali16**	ocular	B	Gambia	1985	ocular	ERR189738	ERS153015	-	Hadfield 2017
**B_Jali20**	ocular	B	Gambia	1985	ocular	-	-	FM872308	Seth-Smith 2009
**B_M48**	ocular	B	Gambia	2007	ocular	ERR175631	ERS177817	-	Hadfield 2017
**B_Tunis864**	ocular	B	Tunisia	1976	ocular	ERR12253485	ERS16770978	-	Ghasemian 2024
**B_TZ1A828_OT**	ocular	B	Tanzania	1998	ocular	-	-	FM872307	Seth-Smith 2009
**Ba_Apache2**	ocular	Ba	USA	1960	ocular	ERR140762	ERS095032	-	Andersson 2016
**C_TW3**	ocular	C	Taiwan	1959	ocular	ERR558499	ERS177778	-	Andersson 2016
**D_SotonD6**	genital	D	UK	2009	endocervix	ERR027328	ERS008762	-	Harris 2012

*ENA: European nucleotide archive

Supplementary Table 3. Baseline demographic characteristics, *Chlamydia trachomatis ompA* genotyping results, and trachoma clinical grades for study participants.

MDA*	Sampling year	Sample ID	Village	Sex	Age start	*omp*A genotype	TF*	TP*	TS*	CO*	TT*
Pre-MDA	1st year	**01_030**	1	Female	5	B	0	0	1	0	0
		**01_031**	1	Female	5	B	3	3	0	0	0
		**01_041**	1	Female	5	B	2	3	0	0	0
		**01_043**	1	Male	4	B	3	3	0	0	0
		**01_107**	2	Female	6	B	3	2	1	0	0
		**01_115**	2	Male	9	B	1	2	1	0	0
		**01_120**	2	Male	4	B	2	1	1	0	0
		**01_174**	2	Female	6	B	3	2	1	0	0
		**01_192**	2	Female	6	B	3	0	0	0	0
		**01_218**	2	Female	5	B	3	2	1	0	0
		**01_250**	3	Female	6	B	3	3	1	0	0
		**01_264**	3	Male	6	B	2	3	0	0	0
		**01_268**	3	Female	11	B	2	2	0	0	0
		**01_403**	3	Male	5	A	3	2	0	0	0
		**01_404**	3	Female	5	A	3	1	0	0	0
		**01_424**	3	Female	7	B	2	3	2	0	0
		**01_456**	3	Female	4	B	2	0	0	0	0
		**02_036**	1	Female	8	B	3	3	0	0	0
		**02_078**	1	Female	10	B	3	3	1	0	0
		**02_134**	2	Male	5	B	0	0	0	0	0
		**02_185**	2	Female	10	B	3	3	0	0	0
		**02_195**	2	Female	6	B	2	3	1	0	0
		**02_337**	3	Male	6	B	3	3	0	0	0
		**02_361**	3	Female	5	A	2	3	1	0	0
		**02_386**	3	Female	8	A	0	0	0	0	0
		**02_415**	3	Female	5	B	3	1	0	0	0
		**02_451**	3	Female	5	B	1	2	0	0	0
		**03_021**	1	Male	5	B	0	0	1	0	0
		**03_175**	2	Female	10	B	0	3	0	0	0
		**03_176**	2	Male	5	B	3	3	0	0	0
		**03_180**	2	Male	10	B	3	3	0	0	0
		**03_209**	1	Male	6	B	3	3	0	0	0
		**03_210**	1	Male	7	B	3	3	0	0	0
		**03_226**	2	Female	7	B	0	0	0	0	0
		**03_248**	3	Male	6	B	3	3	0	0	0
		**03_250**	3	Female	6	B	3	0	0	0	0
		**03_255**	3	Male	5	B	3	3	0	0	0
		**03_373**	3	Female	12	B	1	2	1	0	0
		**03_394**	3	Male	4	B	3	2	0	0	0
		**03_431**	1	Male	10	B	1	2	0	0	0
Post-MDA	2nd year	**04_152**	2	Male	6	B	1	1	0	0	0
		**05_351**	3	Female	4	B	3	1	1	0	0
		**05_356**	3	Female	8	B	1	1	1	0	0
		**05_436**	3	Female	5	B	3	1	0	0	0
		**07_271**	3	Female	6	A	0	0	0	0	0
		**07_313**	3	Female	7	A	0	0	0	0	0
		**07_314**	3	Female	11	A	0	0	0	0	0
		**07_316**	3	Male	7	A	3	2	0	0	0
		**07_321**	3	Male	5	A	2	0	0	0	0
	3rd year	**08_158**	2	Male	8	A	3	1	0	0	0
		**08_290**	3	Male	11	B	3	2	0	0	0
		**09_199**	1	Female	6	A	3	0	0	0	0
		**09_224**	2	Female	10	A	1	1	0	0	0

		**09_448**	3	Male	7	B	2	0	0	0	0
		**10_087**	1	Male	7	A	1	0	0	0	0
		**10_102**	2	Female	6	A	1	2	0	0	0
		**10_119**	2	Male	10	A	3	1	0	0	0
		**10_124**	2	Male	6	A	3	2	0	0	0
		**10_136**	2	Male	7	A	1	2	0	0	0
		**10_155**	2	Female	6	B	1	1	0	0	0
		**10_235**	2	Female	10	A	0	2	1	0	0
		**10_284**	3	Female	9	A	2	2	0	0	0
		**10_256**	3	Male	11	A	1	3	0	0	0
		**10_259**	3	Male	6	A	0	0	0	0	0
		**10_287**	3	Male	6	A	2	2	0	0	0
		**10_400**	3	Male	4	A	0	0	0	0	0
		**11_109**	2	Male	5	B	1	0	0	0	0
		**11_126**	2	Female	8	B	1	0	0	0	0
		**11_187**	2	Female	5	A	2	0	0	0	0
		**11_197**	1	Male	9	A	1	0	0	0	0
		**11_200**	1	Male	10	A	1	0	0	0	0
		**11_208**	1	Female	6	A	1	2	0	0	0
		**11_344**	3	Female	6	A	0	0	0	0	0
		**11_358**	3	Female	8	A	0	0	0	0	0
		**11_418**	3	Male	9	A	0	0	0	0	0
		**11_419**	3	Male	8	A	0	0	0	0	0
	**4th year**	**12_296**	3	Female	8	A	3	1	0	0	0
		**12_353**	3	Female	7	A	3	0	0	0	0
		**12_355**	3	Female	5	A	3	0	0	0	0
		**12_373**	3	Female	12	A	2	0	0	0	0
		**12_376**	3	Female	10	A	2	1	0	0	0
		**13_156**	2	Male	10	A	1	2	0	0	0
		**13_166**	2	Male	7	A	0	0	0	0	0
		**13_169**	2	Male	7	A	3	2	0	0	0
		**13_173**	2	Male	5	A	3	1	0	0	0
		**13_190**	2	Male	8	A	0	1	0	0	0
		**13_191**	2	Male	10	A	0	0	0	0	0
		**13_352**	3	Male	6	A	1	0	0	0	0
		**13_369**	3	Female	9	A	1	3	0	0	0
		**13_379**	3	Female	7	A	1	2	0	0	0
		**13_407**	3	Female	6	A	1	2	0	0	0
		**13_417**	3	Female	9	A	1	2	0	0	0
		**13_420**	3	Male	8	A	2	0	0	0	0
		**14_203**	1	Female	7	A	3	2	0	0	0
		**14_224**	2	Female	10	A	2	1	0	0	0
		**14_227**	2	Female	5	B	3	3	0	0	0
		**14_230**	2	Male	7	A	3	3	0	0	0
		**14_233**	2	Female	9	A	1	0	0	0	0
		**14_235**	2	Female	10	A	3	1	0	0	0
		**14_238**	3	Female	5	A	3	1	0	0	0
		**14_245**	3	Female	6	A	1	2	0	0	0
		**14_249**	3	Female	5	A	0	1	0	0	0
		**14_297**	3	Male	11	A	0	0	0	0	0
		**14_326**	3	Female	8	A	0	0	0	0	0
		**14_352**	3	Male	6	A	1	0	0	0	0
		**14_398**	3	Female	8	A	3	1	0	0	0
		**14_401**	3	Male	4	A	0	0	2	0	0
		**14_408**	3	Male	10	A	3	2	0	0	0
		**15_058**	1	Female	7	B	3	2	0	0	0
		**15_169**	2	Male	7	A	2	1	0	0	0
		**15_182**	2	Male	7	A	3	1	0	0	0

		**15_192**	2	Female	6	A	3	3	0	0	0
		**15_278**	3	Female	6	A	3	0	0	0	0
		**15_283**	3	Female	9	A	2	0	0	0	0
		**15_289**	3	Female	11	A	0	1	0	0	0
		**15_300**	3	Male	8	A	1	1	0	0	0
		**15_307**	3	Female	9	A	3	3	0	0	0
		**15_323**	3	Male	6	A	2	1	0	0	0

*MDA: mass drug administration, TF: trachomatous inflammation-follicular, TP: trachomatous papillary-inflammation, TS: trachomatous scarring, CO: corneal opacity, TT: trachomatous trichiasis

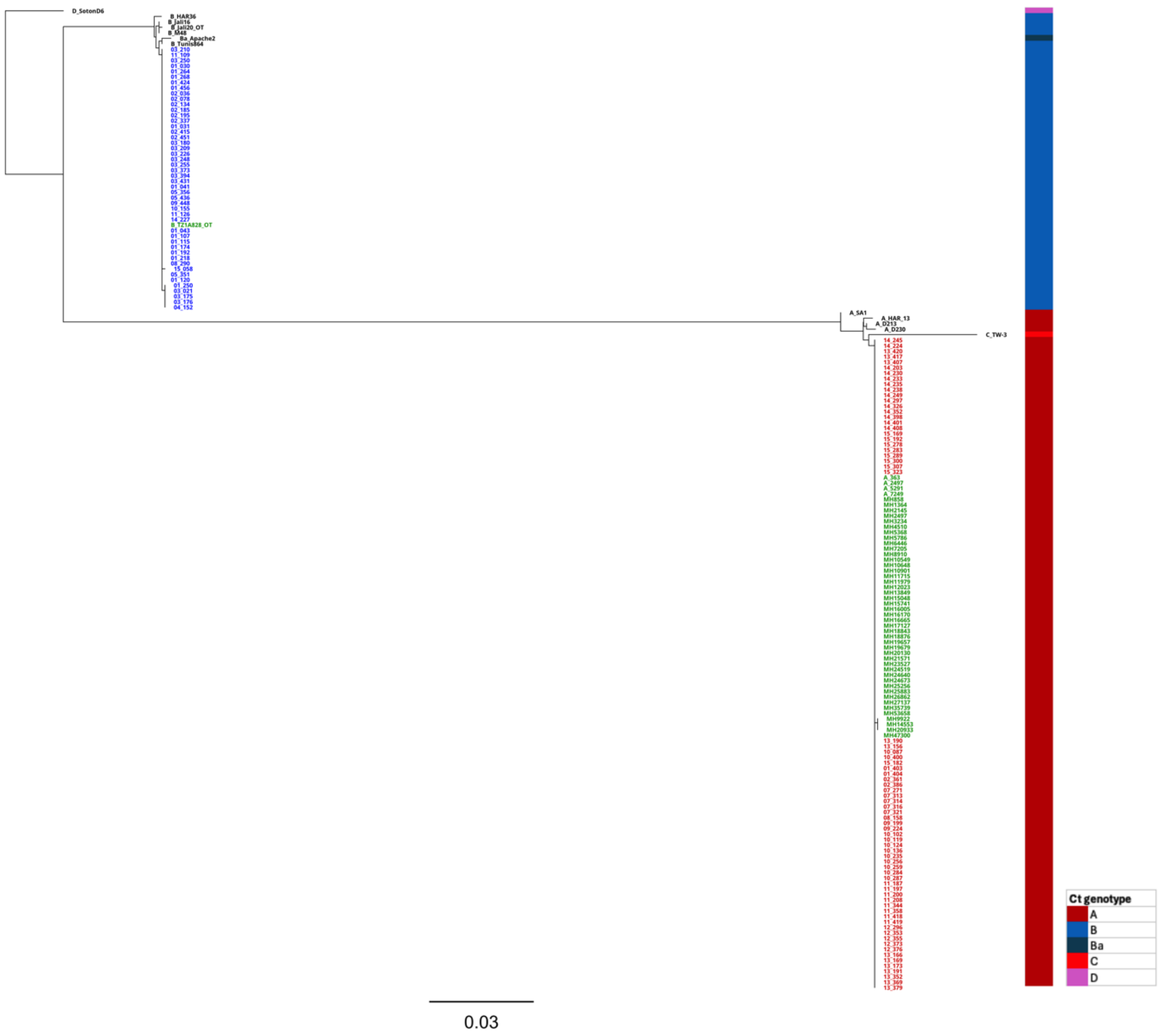

Supplementary Fig. 1. Maximum likelihood phylogenetic reconstruction of ocular *Chlamydia trachomatis* (Ct) *omp*A sequences. The analysis includes *omp*A sequences from 118 Tanzanian strains and 60 trachoma reference strains, with Ct strain D_SotonD6 (T2) as an outgroup. Tanzanian samples from this study tested positive for CtA are denoted in red, while those for CtB are indicated in blue. All reference strains originating from Tanzania represented in green, and other reference strains are in black. The column on the right corresponds to *omp*A genotype for each strain. The scale bar represents the number of nucleotide substitutions per site, indicating evolutionary distance.

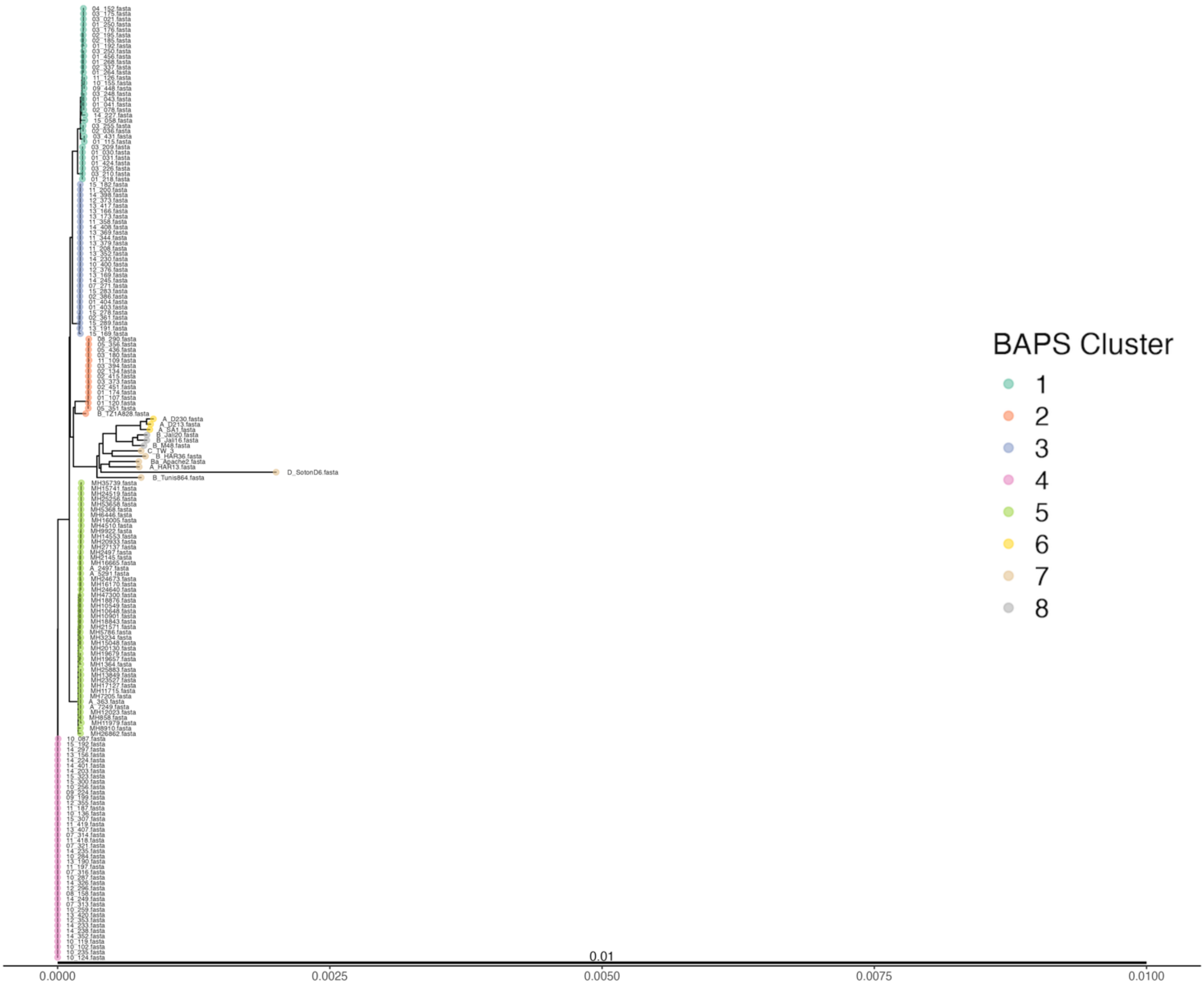

Supplementary Fig. 2. Hierarchical bayesian analysis of population structure (hierBAPS) of *Chlamydia trachomatis* (Ct) whole genomes. The phylogenetic tree illustrates the genetic relationships among 118 Tanzanian Ct strains and 61 Ct reference genomes. The rectangular tree was constructed using the neighbor-joining method based on raw genetic distances. Coloured tip points represent different BAPS clusters identified at level 1 of hierarchical clustering (maximum depth=2, n.pops=50). Clusters are color-coded according to the Set2 colour palette, with tip labels shown in black. Scale bar represents 0.01 nucleotide substitutions per site.

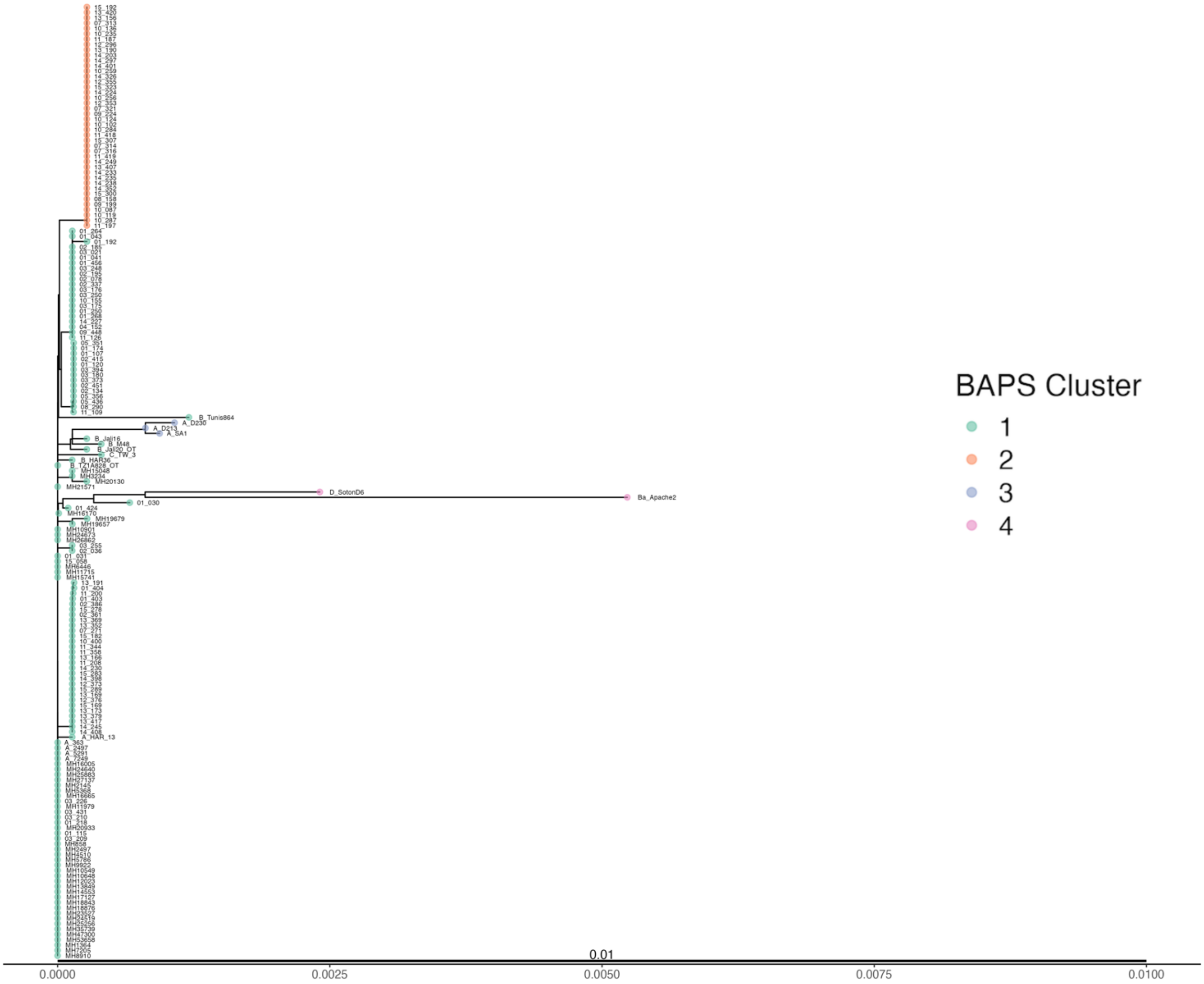

Supplementary Fig. 3. Hierarchical bayesian analysis of population structure (hierBAPS) of *Chlamydia trachomatis* (Ct) plasmid. The phylogenetic tree illustrates the genetic relationships among 118 Tanzanian Ct strains and 61 Ct reference plasmids. The rectangular tree was constructed using the neighbor-joining method based on raw genetic distances. Coloured tip points represent different BAPS clusters identified at level 1 of hierarchical clustering (maximum depth=2, n.pops=20). Clusters are color-coded according to the Set2 colour palette, with tip labels shown in black. Scale bar represents 0.01 nucleotide substitutions per site.

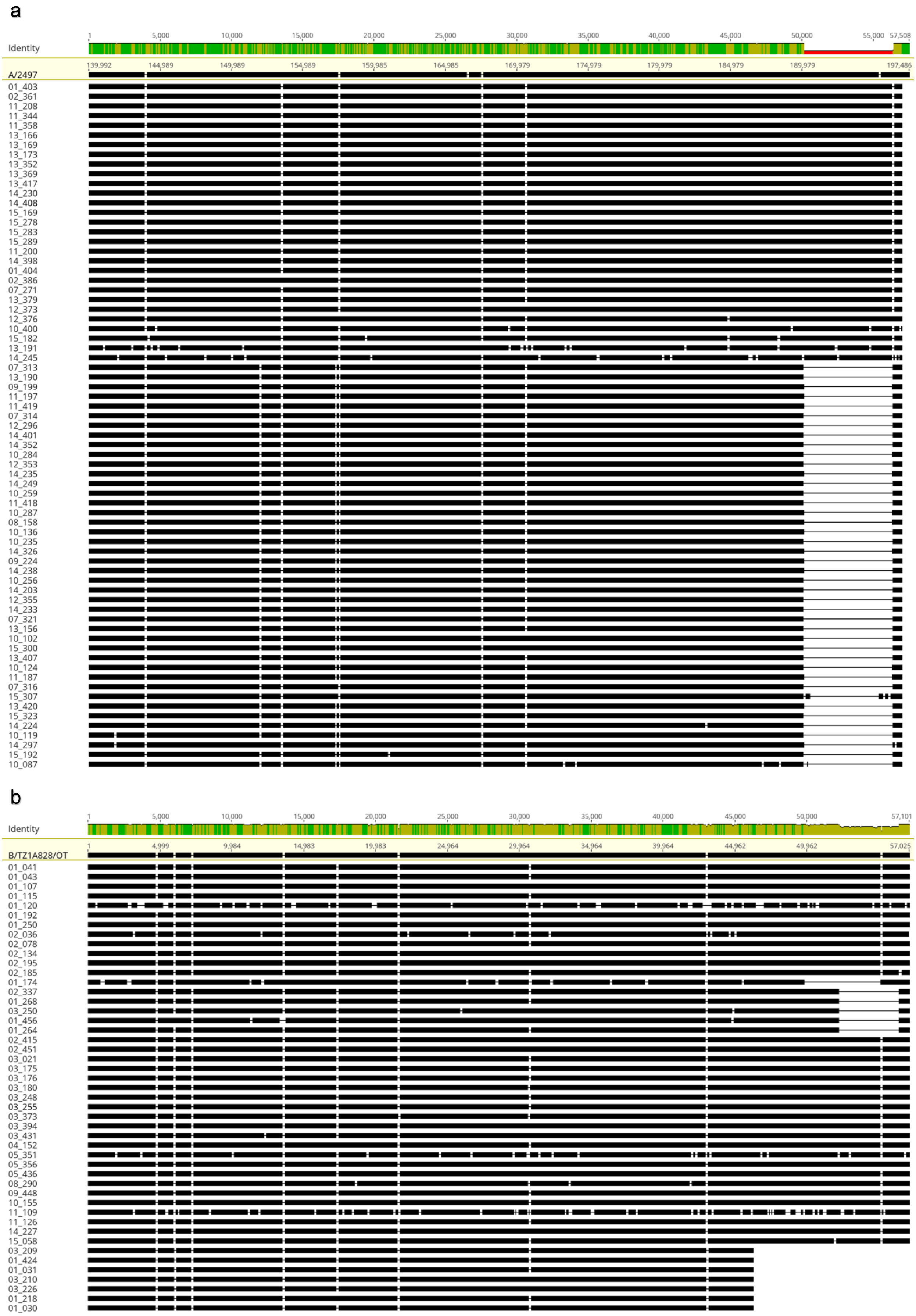

a
Identity
1
5,000
10,000
15,000
20,000
25,000
30,000
35,000
40,000
45,000
50,000
55,000
57,508
139,992
144,989
149,989
154,989
159,985
164,985
169,979
174,979
179,979
184,979
189,979
197,486
A/2497
01_403
02_361
11_208
11_344
11_358
13_166
13_169
13_173
13_352
13_369
13_417
14_230
14_408
15_169
15_278
15_283
15_289
11_200
14_398
01_404
02_386
07_271
13_379
12_373
12_376
10_400
15_182
13_191
14_245
07_313
13_190
09_199
11_197
11_419
07_314
12_296
14_401
14_352
10_284
12_353
14_235
14_249
10_259
11_418
10_287
08_158
10_136
10_235
14_326
09_224
14_238
10_256
14_203
12_355
14_233
07_321
13_156
10_102
15_300
13_407
10_124
11_187
07_316
15_307
13_420
15_323
14_224
10_119
14_297
15_192
10_087
b
Identity
1
5,000
10,000
15,000
20,000
25,000
30,000
35,000
40,000
45,000
50,000
57,101
1
4,999
9,984
14,983
19,983
24,964
29,964
34,964
39,964
44,962
49,962
57,025
B/TZ1A828/OT
01_041
01_043
01_107
01_115
01_120
01_192
01_250
02_036
02_078
02_134
02_195
02_185
01_174
02_337
01_268
03_250
01_456
01_264
02_415
02_451
03_021
03_175
03_176
03_180
03_248
03_255
03_373
03_394
03_431
04_152
05_351
05_356
05_436
08_290
09_448
10_155
11_109
11_126
14_227
15_058
03_209
01_424
01_031
03_210
03_226
01_218
01_030

Supplementary Fig. 4. Alignment view of the plasticity zone (PZ) in Tanzanian *Chlamydia trachomatis* (Ct) sequences. MAFFT was used to align Ct PZ spanning from *acc*B to *trp*A in the Tanzanian sequences. (a) Represents PZ in Tanzanian CtA sequences in comparison to Ct reference strain A/2497. (b) Represents PZ in the Tanzanian CtB sequences in comparison to Ct reference strain B/TZ1A828/OT.

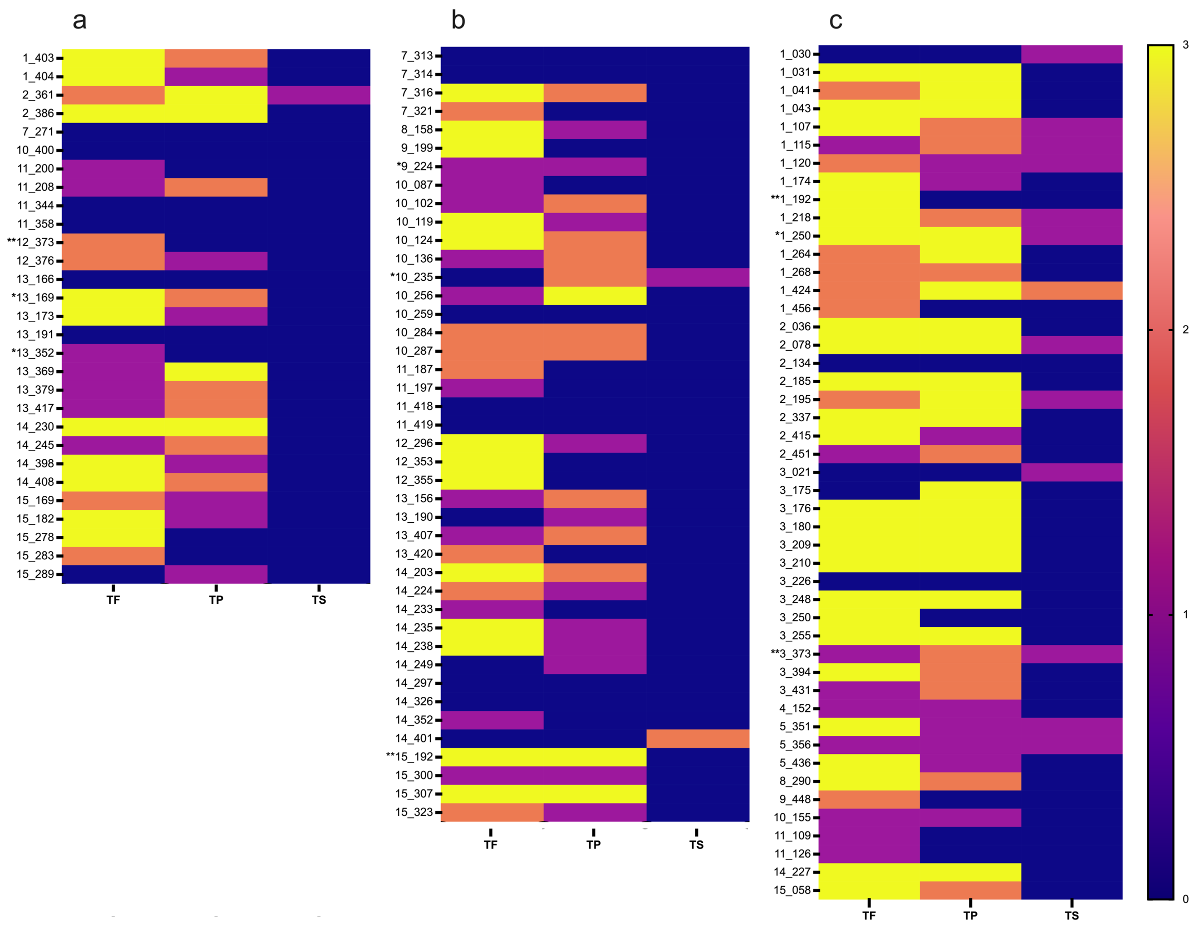

Supplementary Fig. 5. Trachoma grades and scores recorded for each participant on the day of sampling. Heatmap represents trachoma grades: TF, TP, and TS recorded on the day of sampling for individuals grouped as CtA clade 1 (a), CtA clade 2 (b), and CtB (c). The colour gradient bar represents trachoma scores ranging from 0 to 3, linked to the trachoma grades. TF: trachomatous inflammation-follicular, TP: trachomatous papillary-inflammation, and TS: trachomatous scarring
